# EB1 regulates attachment of Ska1 with microtubules by forming extended structures on the microtubule lattice

**DOI:** 10.1038/ncomms11665

**Published:** 2016-05-26

**Authors:** Geethu E. Thomas, K. Bandopadhyay, Sabyasachi Sutradhar, M. R. Renjith, Puja Singh, K. K. Gireesh, Steny Simon, Binshad Badarudeen, Hindol Gupta, Manidipa Banerjee, Raja Paul, J. Mitra, Tapas K. Manna

**Affiliations:** 1 School of Biology, Indian Institute of Science Education and Research Thiruvananthapuram, CET Campus, Thiruvananthapuram 695016, India; 2 School of Physics, Indian Institute of Science Education and Research Thiruvananthapuram, CET Campus, Thiruvananthapuram 695016, India; 3 Department of Solid State Physics, Indian Association for the Cultivation of Science, Kolkata 700032, India; 4 Kusuma School of Biological Sciences, Indian Institute of Technology Delhi, New Delhi 110016, India

## Abstract

Kinetochore couples chromosome movement to dynamic microtubules, a process that is fundamental to mitosis in all eukaryotes but poorly understood. In vertebrates, spindle-kinetochore-associated (Ska1–3) protein complex plays an important role in this process. However, the proteins that stabilize Ska-mediated kinetochore-microtubule attachment remain unknown. Here we show that microtubule plus-end tracking protein EB1 facilitates Ska localization on microtubules in vertebrate cells. EB1 depletion results in a significant reduction of Ska1 recruitment onto microtubules and defects in mitotic chromosome alignment, which is also reflected in computational modelling. Biochemical experiments reveal that EB1 interacts with Ska1, facilitates Ska1-microtubule attachment and together stabilizes microtubules. Structural studies reveal that EB1 either with Ska1 or Ska complex forms extended structures on microtubule lattice. Results indicate that EB1 promotes Ska association with K-fibres and facilitates kinetochore-microtubule attachment. They also implicate that in vertebrates, chromosome coupling to dynamic microtubules could be mediated through EB1-Ska extended structures.

## Abstract (web summary)

Ska1 is a kinetochore-localised protein that couples kinetochore movement to microtubule (MT) depolymerisation. Here Thomas *et al*. show that the MT +TIP binding protein EB1 recruits Ska1 to the MT-kinetochore interface and stabilises the interaction between Ska1 and MTs.

## Graphical abstract

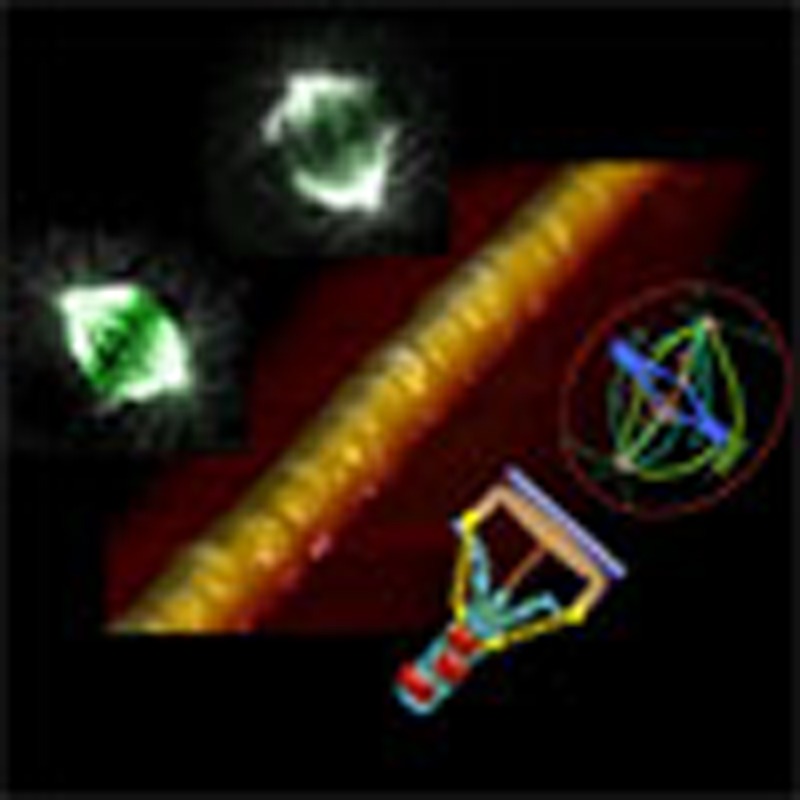

In eukaryotes, mitotic chromosome alignment and segregation require establishment of physical connections between chromosomes and spindle microtubules (MTs). A critical step in this pathway is the regulated attachment of spindle MTs with the kinetochore (KT), a supramolecular complex made up of ∼100 proteins assembled on centromeric chromatin^1^,^2^. Although a plethora of KT- and spindle-associated factors involved in KT–MT (kMT) attachment have been identified in recent years^3^,^4^,^5^,^6^,^7^, the mechanisms by which the KT remains attached to the spindles despite the rapid dynamics of polymerization (growth) and depolymerization (shrinking) of MTs and how the KT couples these fast changing structures to chromosome movements remain unclear.

In budding yeast, the physical connection between KT and MTs is provided by the 10 protein complex, Dam1/DASH^8^,^9^,^10^. Dam1/DASH binds to both MTs and Ndc80, the main outer KT component that interlinks KT with the MT plus ends through its stretchable long coiled-coil structure^8^,^9^,^10^,^11^,^12^,^13^ and coordinates KT movements as the MTs polymerize and depolymerize^9^,^10^,^11^,^12^,^13^. Because Dam1/DASH complex can form dynamic ring structures that can encircle around MT lattice *in vitro*^8^,^14^,^15^, a mechanism that the Dam1/DASH rings can couple KT movement to the polymerizing and depolymerizing MTs *in vivo* by sliding along the MT surface, has been suggested^9^. However, no orthologs of Dam1/DASH proteins exist in metazoans. Therefore, the nature of the structural platform that mediates chromosome/KT processivity in higher organisms has remained elusive. In recent studies, the spindle- and KT-associated protein complex, Ska has been shown to play an important role in coupling chromosome movements with dynamic MTs in vertebrate cells analogous to the function of the Dam1/DASH complex in yeasts^16^,^17^,^18^,^19^,^20^,^21^,^22^.

The Ska complex consists of three components, Ska1, Ska2 and Ska3 (ref. 19). Ska1 knock-down leads to chromosome alignment defects and destabilization of KT-attached MT in human cells^22^. Ska1 can bind simultaneously to Ndc80 (human Hec1) and to MTs, and it couples KT movements to depolymerizing MTs through its attachment with the curved protofilaments of depolymerizing MTs^16^,^22^,^23^. However, because the depolymerizing protofilaments themselves are highly unstable in nature, how Ska1 maintains its stable connection with the MTs during the processive movement of chromosomes and harnesses the forces produced by dynamic MTs remains to be understood. Because a host of additional proteins localize to the KT–MT interface^4^, we hypothesized that other factors are involved in the formation of functional linkages with Ska-mediated KT–MT attachment.

A member of the plus-end tracking proteins (+TIPs), EB1, is an important regulator of MT plus ends in organisms from yeast to human^24^,^25^. EB1 regulates plus-end dynamics^26^,^27^ and is essential for maintaining spindle symmetry and chromosome alignment during mitosis^28^,^29^,^30^. EB1 has been shown to associate with KTs during mitosis through its attachment to the plus ends of mitotic spindles^31^,^32^. However, the molecular details of EB1 interactions at the spindle-KT interface are not clearly known. Here we report that EB1 functions in chromosome alignment by recruiting Ska1 to the spindle–KT interface and stabilizing Ska1 interactions with the MTs. Biochemical analyses indicate that EB1 stimulates Ska1 recruitment onto MTs by forming complex with Ska1 and by imparting stabilization onto MTs. High-resolution atomic force microscopy (AFM) and transmission electron microscopy (TEM) analyses further reveal the distinct structural identities of EB1–Ska1 and EB1–Ska complexes on the MTs. The results demonstrate EB1 as a critical regulator of Ska-mediated MT–KT attachment and provide new insights into the molecular details of MT interactions with spindle-KT proteins in vertebrates.

## Results

### EB1 is essential for Ska1 localization on the spindle MTs

**Figure 1:**
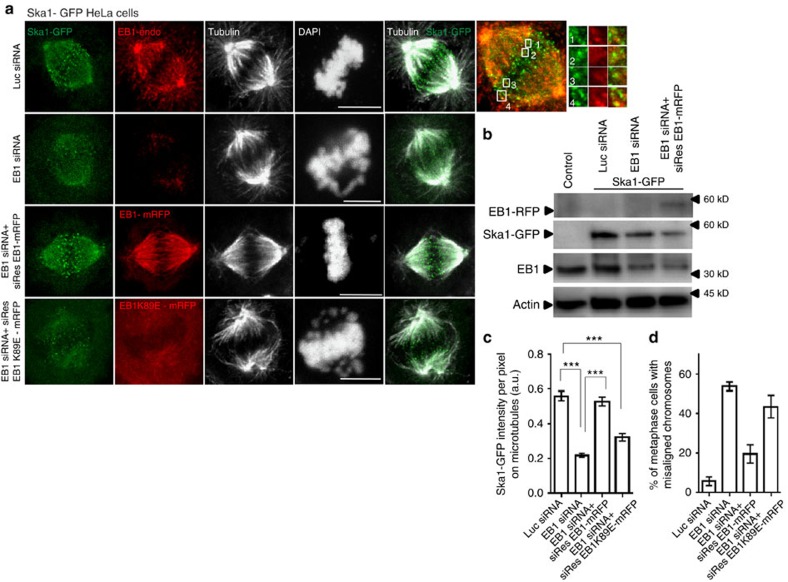
EB1 co-localizes with Ska1 on the spindle microtubules and its depletion induces loss of Ska1 from the microtubules. (**a**) Immunofluorescence confocal images of Ska1–GFP expressing HeLa cells transfected with luciferase siRNA, EB1 siRNA, EB1 siRNA+siRNA-resistant EB1–mRFP construct (siRes EB1–mRFP) and EB1 siRNA+siRNA-resistant EB1K89E-mRFP mutant (siRes EB1K89E-mRFP). Scale bar, 10 μm. Enlarged image on the right shows localization of Ska1–GFP (green) and endogenous EB1 (EB1-endo) (red) in a representative control cell. (**b**) Western blot showing the expression levels of EB1 (endogenous), Ska1–GFP and siResEB1-mRFP in Ska1–GFP HeLa cells under different conditions as specified. (**c**) Quantification of intensity of Ska1–GFP on the spindle microtubules at different conditions as specified, representation of regions selected for quantification is shown in Supplementary Fig. 1H, method of analysis is provided in ‘Methods' section (*n*=∼10–30) ***P<10^−10^. (**d**) Plot shows the percentage of metaphase cells with misaligned chromosomes in Ska1–GFP HeLa cells under different conditions as specified, (*n*=∼100). Data are mean±s.e.m.

Ska1 has previously been shown to associate with both the KT and the spindle MT plus ends^22^. We observed that localization of EB1 was, in many places, similar with that of Ska1 in the mitotic cells, both on the MTs at and near the spindle–KT interface and also at the centrosomes. We visualized localization of the two proteins both in HeLa cells stably expressing Ska1–GFP (green fluorescent protein), hereafter referred as Ska1–GFP HeLa cells and in Cos-7 cells. In the HeLa cells, Ska1–GFP was found to be concentrated as bright dot-like signals at the MT–chromosome interface, presumably at and near the plus ends of the MTs and many of those Ska1–GFP dots had EB1 either co-localized or localized very closely (Fig. 1a). Similar localization pattern was observed in the Cos-7 cells (Supplementary Fig. 1A,B). The correlation coefficients of the two proteins around the metaphase plate was about ∼0.41 and ∼0.42 in Ska1–GFP HeLa and Cos-7 cells, respectively (Supplementary Fig. 1A; Methods section). The spatial positioning of Ska1–GFP and EB1 in a representative HeLa cell are shown in Supplementary Movie 1. It should be noted that in addition to localization at or near the plus ends, the two proteins also appeared to be co-localized on the MTs in the regions further distal from the plus ends (Fig. 1a, Supplementary Fig. 1B). This could due to association of EB1 along the lengths of the MTs in addition to the plus ends as EB1 has been shown to weakly associate with the surface of the MTs in addition to its strong association with the plus end^33^.

As EB1 regulates structural organization of the plus end^34^, the process that is linked with spindle–KT interaction^22^, it is possible that Ska1 localization at the spindle–KT interface is controlled by EB1. To test this hypothesis, we first examined the effect of EB1 depletion on Ska1 localization in both the mitotic Ska1–GFP HeLa and Cos-7 cells. After 48 h of EB1 small interfering RNA (siRNA) transfection, the level of EB1 was reduced by ∼70% in Ska1–GFP HeLa cells and by ∼90% in Cos-7 cells when compared with the respective control cells treated with luciferase siRNA (Fig. 1b, Supplementary Fig. 1C). In both these cells, the localization of Ska1–GFP or Ska1 on the MTs on both sides of the metaphase plate was significantly impaired on EB1 depletion (Fig. 1a, Supplementary Fig. 1B). The intensity of Ska1–GFP or Ska1 on MTs was reduced by ∼60% and by ∼45% in the Ska1–GFP HeLa (Fig. 1c) and Cos-7 cells (Supplementary Fig. 1D,H), respectively. Ska3 was also found to be delocalized from the MTs in EB1-depleted cells (Supplementary Fig. 1F). In both the cell lines, EB1 depletion led to chromosome misalignments in metaphase cells with characteristic scattering on both sides of metaphase plate. ∼50% cells displayed these defects in either Cos-7 or Ska1–GFP HeLa cells (Supplementary Figs 1E,D). Similar phenotypes were observed in the cells treated with EB1 esiRNA (Methods section; Supplementary Fig. 1G).

We then performed rescue experiments to ensure the specificity of this effect. Ska1–GFP HeLa cells were co-transfected with EB1–siRNA and a EB1–mRFP construct, referred as siRes EB1–mRFP that was resistant to EB1 siRNA. EB1 siRNA treatment reduced the expression of endogenous EB1 without interfering with the expression of siRes EB1–mRFP (Fig. 1b). EB1 depletion-induced loss of Ska1–GFP localization on the MTs was almost completely rescued in the co-transfected cells (Fig. 1a,c). Consistently, the resulting chromosome scattering was also significantly rescued (Fig. 1a,d). Only ∼14% cells under this condition showed misaligned chromosomes compared with ∼55% in the EB1-depleted cells (Fig. 1d). Rescue experiments with a siRNA-resistant EB1–GFP construct in Cos-7 cells also showed similar results (Supplementary Fig. 1B–E). We also found that expression of a siRNA-resistant EB1 mutant, siRes EB1 K89E-mRFP, also referred as EB1 MT-binding mutant that does not possess MT-binding ability^32^, failed to rescue the EB1 depletion-induced loss of Ska1–GFP from the MTs and chromosome misalignments in the Ska1–GFP HeLa cells (Fig. 1a,c,d). This indicates that Ska1 recruitment requires EB1 to be associated with the MTs.

We also compared the EB1 depletion-induced phenotype with those of Ska1-depleted and Ska1, EB1 co-depleted cells. The Ska1-depleted Cos-7 cells equally displayed severe scattering of chromosomes around the metaphase plate as of EB1-depleted cells; however, the distribution of chromosomes appeared to be relatively broader around the spindle equator (compare Supplementary Fig. 2A with Fig. 1b). Similar Ska1 depletion-induced phenotype was reported previously in other cell lines^22^. Interestingly, in the EB1, Ska1 co-depleted cells, the phenotype appeared to be considerably more severe with complete misalignment of chromosomes (Supplementary Fig. 2A,B).

**Figure 2:**
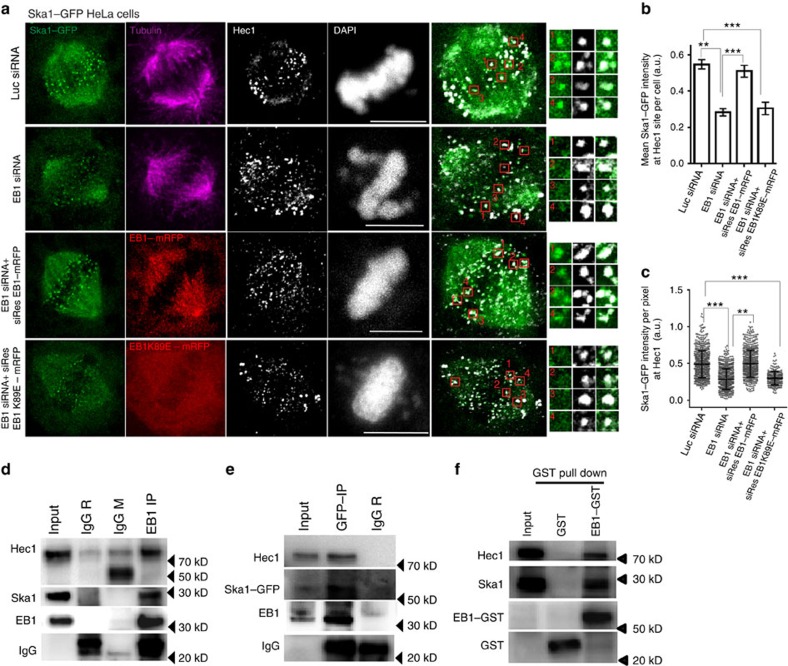
EB1 depletion interferes with Ska1 localization at the spindle-kinetochore attachment site. (**a**) Representative immunofluorescence confocal images of Ska1–GFP HeLa cells transfected with luciferase siRNA, EB1 siRNA, EB1 siRNA+ siRes EB1–mRFP or EB1 siRNA+ siRresEB1-K89E mRFP. Cells were immunostained with α-tubulin (pink) and Hec1 (white) antibodies. DNA was stained with DAPI (shown in white). Scale bar, 10 μm. Enlarged images and the selected regions shown in boxes correspond to Ska1–GFP localization at Hec1 sites at above specified conditions. (**b**) Plot showing quantification of mean Ska1–GFP intensities at Hec1 site considering sum of intensities at all Hec1 sites in a cell at different treatment conditions as specified (no of cells∼10–30). (**c**) Plot showing intensity values of Ska1–GFP at each Hec1 site in individual cells at the specified treatment conditions (data shown for ∼10–30 cells). ***P<10^−10^, **P<10^−5^. Data are mean±s.e.m. (**d**) Lysates of double thymidine-treated synchronized mitotic shake-off HeLa cells were subjected to immunoprecipitation with EB1 antibody followed by Western blot analysis for detecting EB1, Ska1 and Hec1. Rabbit and mouse IgG were used as control. (**e**) Lysate of Ska1–GFP HeLa cells was subjected to immunoprecipitation using rabbit GFP antibody and probed for Ska1–GFP, EB1 and Hec1 in Western blot. (**f**) Lysate of synchronized mitotic HeLa cells was incubated with purified GST–EB1 and subjected to GST pull down using glutathione sepharose beads. Ska1 and Hec1 pulled-down with EB1-GST were detected by Western blot. Purified GST was used as control (shown in middle lane).

We next determined the effect of EB1 depletion on the localization of Ska1 at the spindle–KT attachment sites. For this, we used Hec1 (Ndc80) as an outer KT marker. As shown by a representative image of a control metaphase Cos-7 cell, Ska1 and EB1 were co-localized at the sites of Hec1 (Supplementary Fig. 2C, insets). Quantification of percentage overlap of EB1 with Hec1 and Ska1 with Hec1 were ∼77% and 90%, respectively (Supplementary Fig. 2D). Similar to Cos-7 cells, in the Ska1–GFP HeLa cells, most of the Ska1–GFP bright dots were localized at Hec1 sites (Fig. 2a). On depletion of EB1, the localization of Ska1–GFP at the MT–KT attachment site was substantially impaired (Fig. 2a). Intensity measurements showed ∼40% reduction of the mean Ska1–GFP intensity considering all the Hec1 sites in a cell in the EB1-depleted condition compared with the control (Fig. 2b, Supplementary Fig. 2G). Ska1–GFP intensity at the individual Hec1 sites also showed substantial reduction (Fig. 2c). It was also observed that the loss of Ska1–GFP from the Hec1 sites was rescued when the siRNA-resistant EB1 construct, siRes EB1–mRFP was co-transfected with EB1–siRNA (Fig. 2a–c); whereas, co-transfection of the MT-binding EB1 mutant, siRes EB1K89E-mRFP and EB1–siRNA could not rescue the defects (Fig. 2a–c). Similar phenotypes were observed in Cos-7 cells (Supplementary Fig. 2E,F).

### EB1 interacts with Ska1

Above results suggested that EB1 could mediate interaction with Ska1. Co-immunoprecipitation (Co-IP) experiments with mitotic HeLa cell lysates were performed to determine the interaction. Ska1 was co-precipitated with EB1 (Fig. 2d). Consistent with the known interaction between Ska1 and Hec1, EB1 immunoprecipitate also showed the presence of Hec1. The reverse interactions were confirmed by co-immunoprecipitating Ska1–GFP from the Ska1–GFP HeLa cell lysate (Fig. 2e). The EB1–Ska1 interaction was further confirmed by glutathione *S*-transferase (GST) pull-down assay (Fig. 2f). We also examined MT dependence of these interactions by treating the cells with nocodazole followed by proteasome inhibitor, MG-132. In nocodazole-treated cells, the amount of Ska1 pulled-down with EB1 was reduced by ∼30% and the amount of Hec1 was reduced by ∼70% compared with the cells not treated with nocodazole (Supplementary Fig. 3A), indicating that MTs play important roles in stabilization of these interactions.

### Binding between recombinant EB1 and Ska1

**Figure 3:**
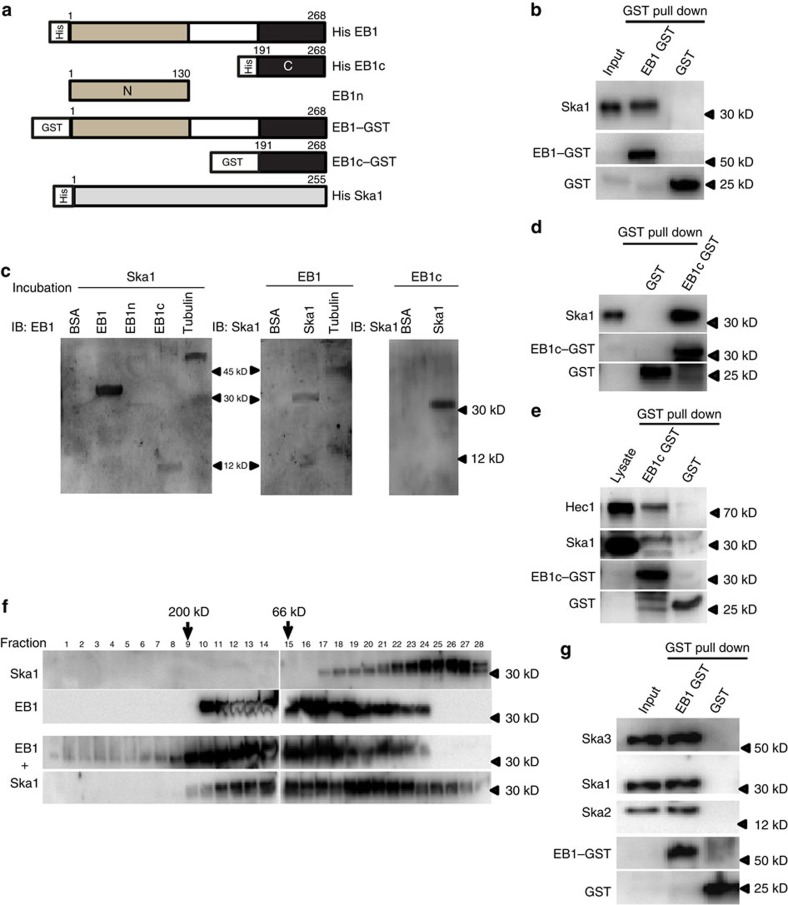
EB1 binds to Ska1 through its C terminus. (**a**) Regions of various deletion constructs of EB1 (C and N terminus) and Ska1 are shown. (**b**) *In vitro* GST pull-down with purified human full length EB1–GST and purified human His-tagged Ska1. Purified GST protein was used as control. (**c**) Far-western blot analysis with immobilized EB1, EB1n and EB1c followed by incubation with purified Ska1. Immobilized BSA and tubulin were used as negative and positive control, respectively. Reverse Far-western with immobilized Ska1 followed by incubation with purified His-EB1 or His-EB1c is shown. Rabbit polyclonal EB1 antibody targeted against the C-terminal region of EB1 was used. It could recognize both the full length EB1and EB1 C terminus. (**d**) The western blot shows *in vitro* GST pull-down using purified EB1c–GST and purified Ska1. (**e**) Lysates of synchronized mitotic HeLa cells was incubated with purified EB1c–GST followed by GST pull down using Sepharose beads and analysed by western blot. (**f**) Purified Ska1 and EB1 or the mixture EB1 and Ska1 was loaded on Superose 12 column and the fractions (200 μl) were analysed by Western blot. Molecular weights of Ska1 and EB1 monomers are 30 and 32 kDa, respectively. (**g**) Western blot image shows GST pull down *in vitro* using purified EB1-GST and purified Ska complex (mixture of Ska1, 2 and Ska3 in 1:1:1 ratio).

We next determined whether EB1 mediates a direct interaction with Ska1. This was accomplished using purified recombinant proteins (Fig. 3a). GST pull-down assay using purified EB1–GST and purified His-tagged Ska1 showed Ska1 association with EB1–GST (Fig. 3b). EB1–Ska1 binding was also confirmed by far-western blot (Fig. 3d) and by *in vitro* Co-IP experiments (Supplementary Fig. 3B). EB1 consists of a MT-binding N-terminal calponin-homology (CH) domain (1–130) (EB1n) and a C-terminal coiled-coil region (191–268) (EB1c) responsible for dimerization and binding to other +TIPs^35^ (Fig. 3a). Both far-western and GST pull-down data showed that EB1 binds to Ska1 through the EB1c domain (Fig. 3c,d). Furthermore, GST pull-down experiment using purified EB1c–GST with the mitotic HeLa cell lysate showed Ska1 being pulled-down with EB1c–GST; and it also showed the presence of Hec1 (Fig. 3e). We next determined whether there is any stable complex formation between EB1 and Ska1. Equimolar mixture of full length purified EB1 and Ska1 were loaded onto size-exclusion column and the eluted fractions were analysed by western blot. Data showed the presence of high-molecular-weight complex between EB1 and Ska1 in the range of ∼100–180 kDa size in addition to the respective dimer and monomer forms of EB1 and Ska1 (Fig. 3f). Interestingly, EB1 when loaded alone also showed formation of oligomers larger than its usual dimer and monomer forms (Fig. 3f). Ska1 was eluted mostly in its monomer form. We also checked the association of the whole Ska complex (Ska1, 2 and 3) with EB1. EB1–GST pull-down with the mixture of all the purified Ska proteins (Ska1, 2 and 3) showed the presence of Ska2 and Ska3 together with Ska1 (Fig. 3g), indicating that the whole Ska complex can associate with EB1.

### EB1 stabilizes MTs by promoting Ska1 association with MTs

**Figure 4:**
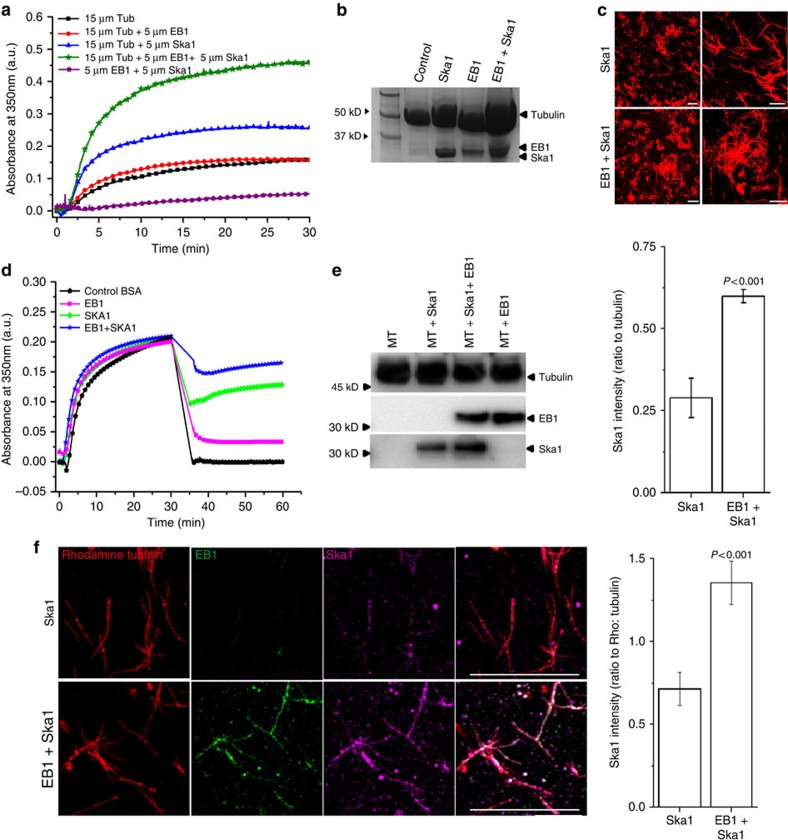
EB1 stabilizes microtubule polymerization together with Ska1 and facilitates Ska1 recruitment onto microtubules. (**a**) Polymerization of tubulin (15 μM) in the presence of either EB1–Ska1 mixture (5 μM each) or EB1 (5 μM) and Ska1 (5 μM) alone was monitored by measuring the turbidity at 360 nm. (**b**) Microtubules polymerized in the presence of EB1–Ska1 mixture or EB1 or Ska1 alone, were sedimented using sucrose cushion and analysed for the proteins present by SDS–PAGE followed by Coomassie staining. (**c**) Images show rhodamine-labelled microtubules polymerized in the presence either EB1–Ska1 mixture (5 μM each) or Ska1 (5 μM) alone. Scale bar, 10 μm. (**d**) Pre-polymerized microtubules with 25% glycerol were incubated with EB1-Ska1 mixture or EB1 and Ska1 alone for 5 min and diluted by 2 fold to measure the stability of microtubules against depolymerization. BSA was used as a control. (**e**) Polymerized microtubules incubated either with EB1 (1 μM), Ska1 (1 μM) or the mixture of EB1 (1 μM) and Ska1 (1 μM) were pelleted by centrifugation through 15% sucrose cushion and the proteins were analysed by western blot. Plot shows the densitometric analysis of the relative amounts of Ska1 recruited to the microtubules in the absence and presence of EB1. (**f**) Rhodamine-tubulin labelled microtubules incubated either with Ska1 (1 μM) or the mixture of EB1 (1 μM) and Ska1 (1 μM) at 35 °C for 5 min were sedimented onto the coverslips through glycerol cushion. Microtubules were immunostained with EB1 (green) and Ska1 (pink) antibodies. Scale bar, 10 μm. Plot depicts the quantification of intensity of Ska1 on the microtubules in the presence and absence of EB1, no of microtubule analysed=∼50. Data are mean±s.e.m.

The class of phenotypes with chromosome misalignment in the EB1-depleted cells may reflect a deregulation of MT polymerization–depolymerization dynamics in response to loss of EB1–Ska1 complex from the MTs. Therefore, we next determined the role of EB1–Ska1 complex on MT polymerization. Purified tubulin was polymerized *in vitro* in the presence of either EB1–Ska1 mixture or individual EB1 or Ska1. EB1–Ska1 complex increased MT polymerization more than two folds compared with Ska1 alone (Fig. 4a). Consistent with previous reports^26^, EB1 alone did not exert any net effect on MT polymerization. The extents of MT polymerization in these different conditions were further confirmed by SDS–PAGE followed by Coomassie blue staining (Fig. 4b). SDS–PAGE data also explained that the increase of turbidity was due to MT polymerization, not due to any mere aggregation of proteins. Fluorescence-based imaging of MTs showed that EB1–Ska1 complex induced MT bundling to a visibly greater extent than Ska1 alone (Fig. 4c). Both the number density and the size of the patches of MT bundles were higher in the presence of EB1–Ska1 complex compared with Ska1 alone (number density: 283±2 (s.e.m.) versus 150±3 (s.e.m.) per mm^2^ considering a threshold size of 250 mm^2^ or above; average bundle size: 1,050±16 μm^2^ versus 660±24 μm^2^). The kinetics of polymerization in the turbidity assay showed a steep increase in turbidity soon after the addition of EB1–Ska1 complex to tubulin, the time when the formation of MT bundles should have been minimum (Fig. 4a). This suggests that the increase of turbidity is primarily due to stimulation of MT polymerization by the EB1–Ska1 complex. Next, we examined the effect of EB1–Ska1 complex on the stability of pre-polymerized MTs. EB1–Ska1 or BSA as control was added to the MTs pre-polymerized by 25% glycerol as an inducer and the reaction mixture was diluted twofold. Control MTs depolymerized almost completely (∼100%) on dilution, whereas the MTs added with EB1–Ska1 mixture depolymerized only ∼40% (Fig. 4d). EB1 or Ska1 alone under similar condition also inhibited depolymerization, but to lesser extents than the EB1–Ska1 mixture. About 60% and ∼90% MTs were depolymerized in the Ska1-treated and EB1-treated conditions, respectively.

We next determined the effect of EB1 on Ska1 recruitment onto MTs. Pre-polymerized MTs were incubated with the mixture of EB1 and Ska1 or Ska1 alone, and the amounts of EB1 and Ska1 associated with the MTs were quantified biochemically. Ska1 association with MTs was significantly increased in the presence of EB1 (Fig. 4e). We next examined the effect of EB1 on Ska1 localization on MTs *in vitro* by using a fluorescence-based assay (‘Methods' section). Mixture of EB1 and Ska1 or Ska1 alone was added to pre-polymerized rhodamine-labelled MTs. A greater (about two folds) amount of Ska1 localized onto MT lattice in presence of EB1 than in its absence (Fig. 4f).

### EB1–Ska1 or EB1–Ska forms extended structures on MT lattice

**Figure 5:**
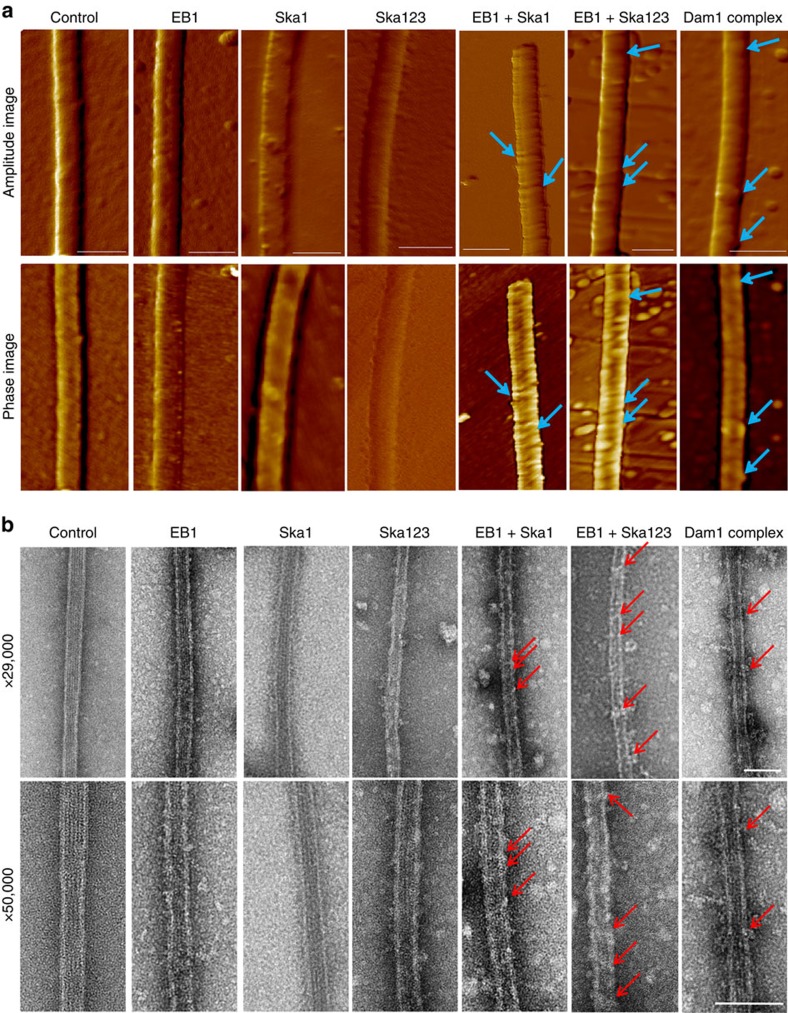
EB1-Ska1 or EB1-Ska (Ska1, 2 and 3) complex forms extended structures on microtubule lattice. (**a**) Representative atomic force microscopy (AFM) images of microtubules in the absence and presence of either EB1, Ska1, Ska (1:1:1 mixture of Ska1, Ska2 and Ska3), mixture EB1 and Ska1 (EB1–Ska1), mixture of EB1 and Ska (EB1-Ska), or Dam1/DASH complex. The amplitude images (upper panel) and phase images (lower panel) of the control microtubules and the microtubules added with the proteins as specified are shown. Scale bar=100 nm. The microtubule-binding structures formed either by EB1-Ska1 or EB1-Ska; or the ring structures formed by Dam1/DASH on the microtubule are shown by white arrows. (**b**) Representative transmission electron microscopy (TEM) images of microtubules with the similar treatment as of **a** acquired at 29 and 50 k magnifications, respectively are shown. The microtubule-binding structures formed either by EB1-Ska1 or EB1-Ska; or the ring structures formed by Dam1/DASH on the microtubule are shown by red arrows. Scale bar, 100 nm. Details of the physical parameters of AFM and TEM images are provided in Supplementary Fig. 4.

To gain insight into the structural morphology of the MTs bound with the proteins, that is, EB1–Ska1, EB1–Ska and the individual EB1 and Ska, we imaged control and the treated MTs using AFM and TEM. Pre-polymerized MTs, incubated with either EB1–Ska1 or EB1–Ska complex, or the individual EB1 or Ska components were drop casted onto mica surface before AFM imaging (see details in ‘Methods' section). Figure 5a (upper two panels) shows the simultaneously acquired amplitude error and phase contrast images of MTs treated with the proteins as indicated, along with that of the control MT. The corresponding topography images are shown in Supplementary Fig. 4. The MTs treated with EB1 showed small globular structures randomly dispersed along the MT lattice (Fig. 5a, Supplementary Fig. 4B). MTs treated only with Ska1 or Ska complex showed isolated interlaced structures extending along the edges (Fig. 5a), consistent with previous reports^23^. Interestingly, addition of EB1 together with either Ska1 or the Ska complex formed binding structures extending along the girth of the MT lattice (Fig. 5a, Supplementary Fig. 4C,D), nearly orthogonal to its axis. In the phase contrast images, the regions decorated by EB1–Ska1 or EB1–Ska showed a distinct contrast indicative of a compositional difference between the decorated structures and the bare MT surface. Under similar conditions, the Dam1/DASH complex-treated MTs showed structures that were reported earlier as ring formations (Fig. 5a)^8^.

Although the MT-binding structures of EB1–Ska1 appear to be similar as of EB1–Ska, the periodicity and the width of these structures showed measurable differences. At comparable protein concentrations, the structures formed by EB1–Ska were relatively close-packed compared with EB1–Ska1 (periodicity (*p*) of 37±3 nm for the former versus 50±5 nm for the later). This characteristic structural morphology is also elucidated in the three-dimensional rendition of topography images (Supplementary Fig. 4C,D). The line scans taken along the MTs are also shown in Supplementary Fig. 4G,H, with the vertical arrows denoting the peak positions of the line scans and with the parameter *p* denoting the periodicity. Thickness (*t*) of the structures, as represented in Supplementary Fig. 4E, formed by EB1–Ska1 or EB1–Ska were estimated by calculating the difference in cross sectional line scans taken across the structures and along the exposed bare MT surface. The structures were found to be of thickness 2±1 nm for those treated with EB1–Ska1 and ∼3±1 nm for those treated with EB1–Ska—the difference presumably accounting for the bulkier nature of the Ska complex as compared with Ska1. The Dam1-DASH rings had the thickness of ∼5±1 nm, which was close to the reported value^8^.

Formation of the structures on the MTs in the presence of EB1–Ska1 or EB1–Ska was further probed by TEM. The panels of Fig. 5b show the negatively stained TEM images of the MTs under different conditions. The average diameter of control MT was ∼30.6±2.9 nm, in good agreement with previously published EM data^8^ (Supplementary Fig. 4I). Similar to the AFM images, TEM images for EB1 showed formation of small but discrete structures on the MT lattice. Both EB1–Ska1 and EB1–Ska mixtures formed extended binding structures that decorated MT lattice in a nearly similar manner like those observed under AFM. As expected, the average breadth (*b*) of the regions decorated with the structures was higher compared with the bare MT (EB1–Ska1: 34.7±3.8 nm; EB1–Ska: 40.3±1.1 nm; Supplementary Fig. 4I). It was also observed that the decorated structures formed by EB1–Ska were relatively wider (*w*) compared with those formed by EB1–Ska1 (EB1–Ska: 15.3 ±1.1 nm; EB1–Ska1: 10.8±1.9 nm), indicating that the structures are modulated in the presence of other Ska components, Ska2 and Ska3. Under similar experimental condition, for the MTs treated with Dam1/DASH complex proteins, the diameter (*d*) and width (*w*) of the Dam1/DASH ring structures were found to be *d:* 54±3.3 nm; *w*: 18.1±2.1 nm (Supplementary Fig. 4I), which were in similar range with the published data^8^. Similar to AFM data, TEM analysis also showed that the Ska complex showed formation of meshwork-like structures on the MT surface, which was consistent with earlier study^23^ (Fig. 5b). Ska1 alone also showed similar structures, although the structures appeared to be smaller than those of the Ska complex.

### Computational model replicates cellular phenotypes

**Figure 6:**
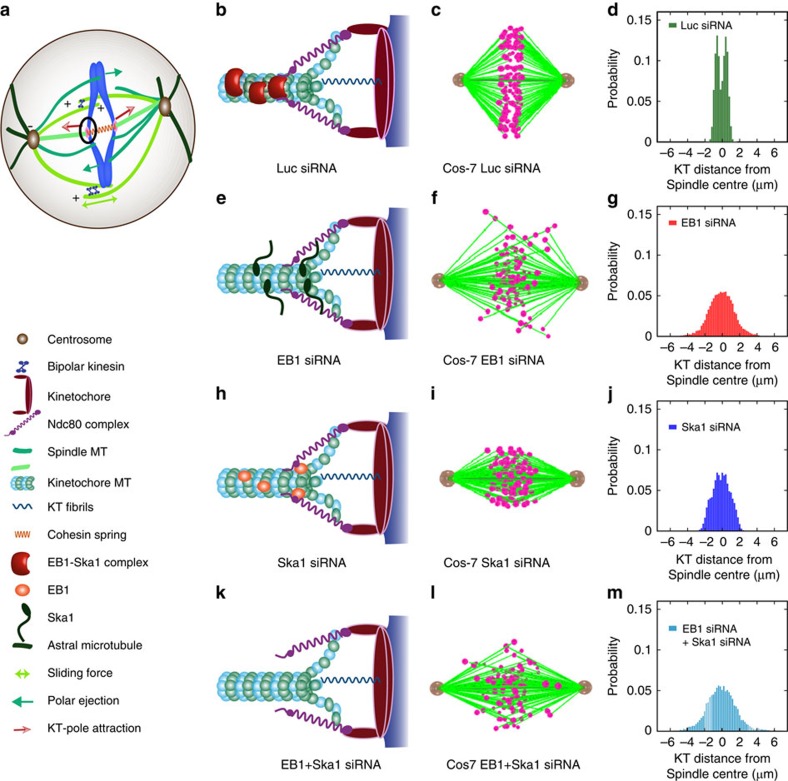
Computational modelling of chromosome distribution. (**a**) Cartoon showing a simplified mechanistic model of mitotic spindles with various components and forces. (**b**) Model of the kMT-KT attachment configuration in control Luc siRNA-treated Cos-7 cells; the model shows that EB1–Ska complex microtubule-binding structures couple kMT with Ndc80 in control cells. (**c**,**d**) are simulation snapshot of chromosomal configuration and distribution around the metaphase plate in these cells, respectively. The snapshots of *in silico* phenotypes show perfectly aligned chromosomes at the metaphase plate. (**e**–**g**) are the model of the kMT–KT attachment, simulation snapshot of chromosomal configuration and distribution in EB1-depleted cells, respectively. In the EB1-depleted cells, due to absence of EB1–Ska structures, the depolymerizing kMTs often fail to recover leading to defective kMT–KT attachment. The *in silico* snapshot shows dispersed chromosomes around the metaphase plate. Note that some residual Ska proteins are still present on the kMT that can weakly interact with Ndc80. (**h**–**j**) are the model of the kMT–KT attachment, simulation snapshot of chromosomal configuration and distribution in Ska1-depleted cells, respectively. The snapshot shows dispersed chromosomes with wider distribution around the metaphase plate. Some dispersed oligomeric structures of EB1 present on the microtubule are able to weakly interact with Ndc80. (**k**–**m**) correspond to the model, chromosomal configuration and distribution when EB1 and Ska1 are co-depleted. The snapshot and the distribution plot show chromosomes scattered all over the spindles. Simulation was carried out with ∼100 samples in each case.

To apprehend the mechanism of kMT attachment based on our experimental observations, we developed a simplified mechanistic model comprising of crucial spindle elements (as shown in Fig. 6a–c): centrosomes, KT, kMT, inter-polar MT, cytoplasmic/astral MTs and various molecular motors (see ‘Supplementary Methods' for details). The attachment between kMT and KT is mediated through the spring-like Ndc80 (Hec1) complex. As illustrated in Fig. 6b, the Ndc80 complex, at one end, interacts with the KT through the KMN network^36^,^37^ and at the other end; it binds with the kMT through EB1–Ska complex as supported by our experimental data. A depolymerizing kMT applies tension on the KT towards the pole through Ndc80 (Hec1) and the EB1–Ska complex that interacts with it. At the same time, the stably attached kMT also experiences a pulling force toward the KT due to stabilization of Ndc80-Ska interaction through EB1–Ska1 binding. This force can facilitate rapid rescue of the kMT from the depolymerizing state to the polymerizing state thereby stabilizing kMT–KT attachment (Fig. 6b). On EB1 deletion, recruitment of Ska complex is disrupted following which Ndc80 couples weakly with the kMT. An impaired kMT–KT attachment fails to generate tension required for rescuing a depolymerizing kMT (Fig. 6e). Moreover, a large body of evidences suggest that the presence of EB1 at the plus end stabilizes MTs from undergoing catastrophe^38^,^39^. Therefore, the effect of EB1 depletion should also include an increased depolymerization. Using suitable parameters (Supplementary Table 1) for the cell lines, Cos-7 and HeLa, *in silico* models were constructed to validate the experimental data. Spindle phenotypes with Luc siRNA for these cell types presented in Fig. 6b,c and Supplementary Fig. 5, showed perfect bipolar spindle morphology with a compact metaphase plate. The distribution of chromosomes in these cells is shown in Fig. 6d. In the absence of EB1, compactness of the metaphase plate was lost and a signifiacnt fraction of the chromosomes were highly misaligned around the spindle equator (Fig. 6e–g), supporting our experimental results (Fig. 1). Moreover, KT distribution measured from the centre of the spindle showed high dispersion in the absence of EB1 compared with the control Luc siRNA-treated cells (compare Fig. 6d with Fig. 6g). Similar results were obtained from the experimental data of Ska1–GFP HeLa cells (Supplementary Fig. 5). In the Ska1-depleted Cos-7 cells, the dispersion of chromosomes was equally severe to that of EB1-depleted cells with a broad distribution of chromosomes around the spindle equator (Fig. 6h–j). However, in the absence of both EB1 and Ska1, chromosomal dispersion was much severe with loss of the metaphase plate (Fig. 6k–m). The spatio-temporal visualization of the phenotypes is presented in the Supplementary Movies of Cos-7 cells (Supplementary Movies 2–5).

## Discussion

The alignment and segregation of mitotic chromosomes during mitosis require strong yet flexible connections between KTs and spindle MTs. The attachments of KTs with both polymerizing and depolymerizing MTs seem to be necessary for harnessing the forces generated at the attachment site. Primarily, the Ska complex has been shown to facilitate chromosome processivity through its interaction with depolymerizing MTs. However, Ska complex promotes the formation of curved protofilaments^16^, a process that is likely to facilitate further depolymerization of the MT lattice and should destabilize KT–MT attachments. Therefore, a mechanism that can impart stabilization to the MT lattice proximal to the depolymerizing ends, seems to be necessary for maintaining persistent attachments of KTs with MTs. Previously, EB1 has been shown to stabilize MT plus-end dynamics presumably through its association with straight MT protofilaments^27^,^34^,^40^,^41^, the structures that represent both the polymerizing end and the lattice proximal to the depolymerizing end^42^,^43^. MT dynamics is considered as the major driver of chromosome movements during mitosis^44^,^45^. Loss of EB1 from the polymerizing end may alter the dynamics and stability of the plus ends of KT-attached MTs (K-fibres), the process that can delocalize the proteins that interlink the KT with MTs. Supporting this possibility, we found that EB1 depletion disrupts the localization of Ska1 on the MTs and at the MT–KT interface (Figs 1 and 2). As Ska complex is crucial for stable attachment of K-fibres with the outer KT protein such as, Ndc80, a likely possibility is that the chromosome misalignment in response to EB1 depletion is a result of the loss of Ska from the MTs. Because EB1 directly interacts with Ska1 (Fig. 3), it could be possible that this interaction stabilizes the dynamics of K-fibres. Supporting this hypothesis, we have shown that EB1–Ska1 complex stabilizes MTs against depolymerization to a considerably greater extent than the individual EB1 or Ska1 protein (Fig. 4). The EB1–Ska1/Ska complex-mediated formation of extended binding structures on the MT lattice (Fig. 5) further suggests that a potential linkage between the spindles and the KT could be mediated through these structures. As Ska1 has been known to bind Hec1 (Ndc80)^23^,^46^, a possible model could be that the extended binding structures of EB1–Ska provide a stable platform that helps maintain persistent attachment of the outer KT with the MTs. In the absence of this platform, efficiency of Ndc80 to hold the K-fibres is compromised as shown in the model (Fig. 6). In the absence of either EB1 or Ska1 and conseqeuntly the large structural platform, the meshwork-like small structures of Ska or the small globular structures of EB1 could still impart some stabilization and provide a moderate support to align the chromosomes. Consistent with this mechanism, we found that in either EB1-depleted or Ska1-depleted cells, a fraction of chromosomes could still manage to align along the metaphase plate, although they appeared to be dispersed or loosely packed (Fig. 1a,d, Supplementary Fig. 1B,E). As Ndc80 has also been shown to interact independently with Ska1 and EB1 (refs 16, 47), it is possible that this partial retaining of chromosomal organization is achieved by interaction of Ndc80 with the Ska structures, when EB1 is absent or with the EB1 structures, when Ska1 is absent. In the absence of both EB1 and Ska1, almost no structural support is present on the MT that can hold Ndc80 and therefore, a more severe defect in K-fiber-KT attachment is likely to result. In support of this, the cells co-depleted of EB1 and Ska1 showed complete misalignment of chromosomes (Supplementary Fig. 2A,B). To this end, we further developed a computational (qualitative) model, which explores the stability of the kMT–KT attachment based on the interaction of Ndc80 complex with the kMT in the absence of either EB1 or Ska1 or both. Our model predicted that loss of EB1-Ska1 interaction weakens Ndc80-mediated kMT–KT attachment, which in turn leads to increased dispersal of KTs/chromosomes on both sides of the metaphase plate (Fig. 6f,g).

Previous studies have shown that the whole Ska complex (Ska1–Ska2–Ska3) forms small meshwork-like pattern and has the ability to move along the depolymerizing MT *in vitro*^22^,^23^. Our AFM and TEM data also showed similar structural pattern of the Ska complex. However, when complexed with EB1, either Ska1 or the whole Ska forms characteristic extended binding structures decorating the MT lattice. It could be possible that the transition from the meshwork-like to these extended structures is tightly regulated in cells and EB1 is involved in inducing such transition. A recent study with EB family protein, EB3 has shown that it decorates MT lattice by attaching along the longitudinal interface of MT^48^. Both our AFM and TEM data show that EB1 decorates the MT surface in the form of discrete patches characteristic of small oligomers (Fig. 5 and Supplementary Fig. 4). Presence of EB1 oligomers larger than its usual dimer form was also apparent in our size-exclusion analysis (Fig. 4). It is possible that these patches can act as nucleating centres for recruiting Ska proteins and transforming their meshwork-like arrangements to the extended structures. In support of this possibility, we showed that EB1 facilitates Ska1 recruitment onto the MTs (Fig. 4). Although both EB1–Ska1 and EB1–Ska form extended MT-binding structures, the structures formed by EB1–Ska are more regular and wider with improved repeatability (Fig. 5, Supplementary Fig. 4). It indicates that the proteins Ska2 and Ska3 have distinct roles in modulating the EB1–Ska1 structures.

The yeast Dam1/DASH complex functions as a stabilizer of MTs and promotes MT growth^14^. Similar to the Dam1/DASH complex, here we show that the EB1–Ska1 complex stimulates MT polymerization, stabilizes MTs against depolymerization and bundles MTs (Fig. 4). The Dam1/DASH complex, which consists of ∼10 protein components, oligomerizes into ring structures that encircle the MT lattice^15^. However, Dam1/DASH complex components cannot form rings independently in the absence of MTs^14^. Similarly, we observed that formation of the MT-binding structures by either EB1–Ska1 or EB1–Ska complex occurs only on MTs. However, despite these similarities, we see distinctive differences between these structures compared with Dam1/DASH rings. Most notably, unlike the Dam1/DASH rings, the EB1–Ska1/Ska structures appear to be very closely associated with the MT lattice. With such closely packed arrangement, it is unlikely that EB1-Ska1/Ska structures can slide along the MTs like the Dam1/DASH rings. Since EB1 is a plus-end tracker and our present data show it as a strong binder of Ska1/Ska complex, it is reasonable to think of a model in which EB1 provides a platform to anchor Ska complex at the MT plus end and to move it together with the dynamic movement of the plus end in the form of the MT-binding structures. This is possible as Ska association with the MTs is increased significantly when EB1 is present than in its absence (Fig. 4e,f).

EB1 has been shown to interact with numerous +TIPs through their conserved SxIP motifs^49^. Binding of the SxIP motifs with the C-terminal coiled-coil domain of EB1 has been shown to be critical for regulation of the plus ends^49^. Analysis of Ska1 sequence across organisms shows that Ska1 consists of an identical motif, SxLP in its N terminus, which is conserved in many vertebrates (Supplementary Fig. 6). Similar to EB1-mediated recruitment of +TIPs proteins, Ska1 recruitment to the plus ends could be mediated through a similar mechanism. Consistent with this idea, we showed that Ska1 interacts with EB1 through the same C-terminal region that interacts with the SxIP motifs of other +TIPs (Fig. 3).

The mechanism of spindle–chromosome attachment is likely to be a highly conserved process across organisms. The MT-binding structures formed by EB1–Ska1 or EB1–Ska complex shown in this study suggest a hypothesis that MT-KT attachment is uniformly conserved in eukaryotes, and this is presumably driven by large oligomeric structural platforms on the MTs. However, the exact molecular architecture of the platform may be different across species. Future studies will be needed to determine the functional relevance of EB1–Ska1/Ska MT-binding structures *in vivo* and also the role of other +TIP proteins in that process.

## Methods

### Reagents and antibodies

DAPI, GTP, PIPES, EGTA and thymidine were obtained from Sigma (St Louis, MO). Dulbecco's modified Eagle's medium (DMEM) supplemented with fetal bovine serum (FBS) was purchased from HiMedia, Mumbai, India. Rhodamine-labelled tubulin was obtained from Cytoskeleton, Inc. (Denver, CO). Mouse monoclonal antibodies against EB1 (Cat # 610534) and Hec1 (Cat # SAB2702380) were obtained from BD Biosciences (California, U.S.A) and Sigma (St Louis, MO), respectively; rabbit polyclonal antibodies of Ska1 (Cat # ab118586), Ska2 (Cat # ab91551), and rat monoclonal antibody of EB1 (Cat # ab53358) were obtained from Abcam (Cambridge, MA, USA). Mouse monoclonal antibody against α-tubulin (Cat # T6199) and the rabbit polyclonal anti-EB1 (Cat # E3406) and anti-GST (Cat # G7781) were obtained from Sigma (St Louis, MO). Monoclonal mouse antibody against Ska3 (Cat # sc-390326) and Hec1 (Cat # sc-135934) were obtained from Santa Cruz Biotechnologies, CA, USA. GFP antibody (Cat # 632381) was obtained from Clontech, Takara (USA). The dilutions of the primary antibodies were: EB1 (IF and WB: 1:500), α-tubulin (IF and WB: 1:600), Ska1 (IF:1:250, WB: 1:500), Hec1 (IF:1:200 (Santa Cruz), WB: 1:500 (Sigma)). FITC, TRITC and peroxidase conjugated secondary antibodies were obtained from Jacksons ImmunoResearch (PA, USA).

### Cell culture and transfection

Cos-7 and HeLa cells originally obtained from ATCC were cultured in DMEM media, supplemented with 10% FBS, 2 mM L-glutamine, 1.5 mg ml^−1^ sodium bicarbonate, 100 μg ml^−1^ penicillin and 100 μg ml^−1^ streptomycin. To make Ska1–GFP expressing stable cell line, pIC291 Ska1–GFP plasmid (Addgene, USA) was transfected in HeLa cells and the Ska1–GFP-expressed cells were selected by growing the cells in selectable marker blasticidin (2 μg ml^−1^) 48 h post tranfection^50^. To suppress protein expression, we used single siRNA or ribonuclease III-prepared siRNA pools (esiRNA)^51^. esiRNA consists of enzymatically prepared siRNA pools comprised of a heterogeneous mixture of siRNAs that target the same mRNA sequence of the gene^52^. Cells at ∼50% confluence were transfected with either EB1 siRNA (5′- AAGUGAAAUUCCAAGCUAAGC -3′) or Ska1 siRNA (5′- GGACUUACUCGUUAUGUUA -3′) (Dharmacon, USA) or EB1 esiRNA (Cat no: EHU045671-Sigma-Aldrich)^53^ targeted against the 331–807 nucleotide region of EB1 (NM_012325.2). Luciferase siRNA (5′- GCCAUUCUAUCCUCUAGAGGAUG -3′) or esiRNA (Cat no: EHUFLUC-Sigma-Aldrich) was used as control. For rescue experiments, siRNA-resistant EB1-GFP (siRes EB1–GFP) or EB1–mRFP (siRes EB1–mRFP) was co-transfected with EB1–siRNA. siRNA-resistant EB1 K89E-mRFP (siResEB1K89E-mRFP) construct was made by site-directed mutagenesis^54^. Lipofectamine (Invitrogen, USA, Life Technologies) was used as vehicle for transfection of siRNA or plasmid DNA.

### Plasmids and proteins

pEGFP-EB1 was used as template for PCR amplification of the coding sequence of the human *EB1* (NM_012325.2) gene. The amplified product was ligated into the pET28a (Novagen, Madison, WI, USA) or pGEX 5 × 3 for 6-His tag or GST-tagged EB1 full length and EB1c (191–268). The plasmid containing EB1n (1–130) without tag was obtained from Michel O. Steinmetz at PSI, SW and the protein was purified by ion exchange chromatography^55^. pEC-S-CDF-His Ska1 and pEC-M-HT-His Ska3 were gifts from A. A. Jeyaprakash, Wellcome Trust Centre for Cell Biology, University of Edinburgh, Edinburgh. pIC317-2 6XHis Ska1–2 co-expression plasmid was kindly provided by Ian Cheeseman, Whitehead Institute, MIT. For making Ska complex, Ska1–2 purified from the co-expression plasmid and purified Ska3 were mixed in 1:1 ratio. The plasmids for Dam1/DASH complex proteins, PC4_3spc34h and PC4_3dad1h were obtained from Simon Jenni, Harvard University. The plasmids containing spc34 and dad1 with 6xHis-tag were expressed under induction of arabinose^8^. All the His-tagged and GST-tagged proteins were expressed in *Escherichia coli* BL21-DE3 cells and purified by Ni^2+^-NTA (QIAGEN) and glutathione Sepharose (GE Healthcare), respectively^56^. Tubulin was purified from goat brain by repetitive cycles of polymerization and depolymerization^56^. Protein concentrations were estimated using the Bradford method using BSA as standard^57^.

### Immunofluorescence microscopy and image analysis

Cells after fixing in methanol at −20 °C were washed with PBS containing 2% bovine serum albumin and 0.5% Triton X-100. The cells were then incubated with primary antibody for 1 h followed by incubation with secondary antibody and DAPI for 45 and 1 min, respectively. Coverslips were mounted using ProLong Gold (Invitrogen), and the images (63 × ) were captured using a Leica SP5 laser confocal microscope. The intensity per pixel of Ska1 or Ska1–GFP localized on the spindle MTs or at the site of Hec1 was quantified by selecting regions of interest (ROI) in each cell after background subtraction using Leica LAS AF Lite software. Representative ROIs selected for the analyses in Ska1–GFP HeLa cells are shown in Supplementary Figs 1H, 2G. Colocalization analysis was performed using Metamorph software (version 7.8.2). The degree of correlation on the colocalization of proteins in the selected regions (as shown by representative images in Supplementary Fig. 1A) was measured in the form of correlation coefficient (ratio of total intensity of colocalizing pixels in the two channels and the total intensity of pixels above threshold in the respective channels)^58^. The overlap percentage of proteins was measured from the ratio of the intensities of the localized proteins in the selected ROIs in the respective channels (as shown in Supplementary Fig. 2D).

### Co-IP and GST pull-down assays

Cells were mitotically synchronized using double thymidine block. EB1 was immunoprecipitated from the cell lysates using the mouse monoclonal EB1 antibody. For IP of Ska1–GFP, Ska1–GFP HeLa cells were lysed and incubated with GFP antibody followed by addition of protein G agarose beads. For *in vivo* GST pull down assay, cell lysates were incubated with full length EB1–GST or EB1c–GST or free GST pre-incubated with glutathione Sepharose beads. *In vitro* GST pull-down was performed by incubating purified proteins either with GST (control) or EB1–GST, or EB1c–GST protein pre-incubated with glutathione Sepharose beads. The beads were washed with lysis buffer and then boiled in SDS–PAGE sample buffer for immunoblot analysis.

### Far-western blot analysis

Equimolar amounts of purified His-tagged proteins were immobilized on SDS–PAGE and transferred to polyvinylidene difluoride membrane. Membrane was blocked with 5% non-fat milk followed by incubation with the interacting protein followed by washing. Membrane was probed with primary antibody against the incubated protein followed by horseradish peroxidase-conjugated secondary antibody and developed for immunoblot using Immobilon reagent (Millipore, USA). Immunoblots were imaged using ChemiDoc XRS System (Bio-Rad, Hercules, CA, USA). BSA was used as a negative control. The uncropped scans of important blots are provided in the Supplementary Information (Supplementary Fig. 7).

### MT polymerization assay

Tubulin (15 μM) and the desired concentrations of proteins (EB1, Ska1, or mixture of EB1 and Ska1) were polymerized in BRB80 buffer (80 mM PIPES, 1 mM EGTA and 1 mM MgCl_2_), pH 6.9 containing 1 mM GTP and 25% glycerol. Polymerization was monitored by measuring the turbidity of the reaction mixtures at 360 nm^59^. The proteins of the reaction mixtures after 30 min of polymerization were pelleted and were subjected to SDS–PAGE to visually assess the amount of MTs formed in these different treatment conditions. To assess EB1-dependent Ska1 recruitment onto MTs, the mixture of EB1 and Ska1 or Ska1 alone was added to the MTs polymerized in presence of 10% dimethylsulphoxide. For quantitative estimation of the MT-bound proteins, the pre-polymerized MTs were incubated with the proteins for 5 min and were passed through 15% sucrose cushion by centrifugation at 100,000*g*. The pellets were re-suspended in cold buffer, and the proteins were loaded onto 10% SDS–PAGE and then subjected to Western blot. Densitometric analyses of the blots were performed using the Quantity one software (Bio-Rad). For visual inspection of the MTs polymerized under the conditions mentioned above, the polymerization reaction was performed using rhodamine-labelled tubulin in 1:12 ratio with unlabelled tubulin. The MTs after fixing with 1% glutaraldehyde were sedimented onto poly-lysine coated glass coverslips via centrifugation through 15% glycerol cushion and were imaged by confocal microscope^59^. The intensity per pixel of Ska1 bound to MTs was quantified by selecting ROI (∼50) of same size on the MT in the Ska1 versus rhodamine-tubulin channel with background reduction using Leica LAS AF Lite analysis software.

### Size-exclusion Chromatography

Purified EB1, Ska1, or the mixture of EB1 and Ska1 were loaded onto a Superose 12 column (Akta 10, GE Healthcare) equilibrated and run at 4 °C. The eluted proteins were collected in fractions of 200 μl each, with 20 μl of each fraction subsequently loaded onto SDS-polyacrylamide gels and analysed by western blot.

### Atomic force microscopy

Tubulin (10 μM) was polymerized at 35 °C for 5 min in BRB80 (80 mM PIPES, 1 mM EGTA and 1 mM MgCl_2_) buffer at pH 6.9 with 1 mM GTP and 10% dimethylsulphoxide. The proteins (EB1, or Ska1/Ska complex or the mixtures of EB1 and Ska1/Ska complex) were added to the MTs and incubated for 5 min at 35 °C. The protein-added or the control MTs were then layered onto freshly cleaved mica sheets and air dried at room temperature. The AFM images were obtained using an ambient AFM (Bruker Multimode 8 using NanoScope V), operated in the tapping mode with Si Cantilevers of force constant∼45 N m^−1^ (MikroMasch HQ:NSC16) and resonant frequency∼190 kHz. Standard topography, amplitude error and phase images were recorded simultaneously to probe the morphology of the MTs and the bound proteins at a minimum of 10 sites across each sample and at various scan ranges varying from 500 nm to 10 μm. It has been known that phase contrast imaging^60^,^61^ offers enhanced two-dimensional surface resolution, not afforded by the other AFM techniques, which is amply evident in the data presented here. Supplementary Fig. 4 shows AFM topography images on bare MTs and those treated with various proteins. The topography images of bare MTs show a reduced apparent height ∼10 nm, which is a consequence of the MT to substrate fixing process^62^,^63^, as demonstrated in earlier studies. The phase image was generated by mapping the phase lag between the external excitation of the oscillating cantilever and its actual response, where the mapped contrast indicates variations in the tip-surface interaction (adhesion, across the surface. Consequently, phase images offer higher resolution compositional material contrast which goes undetected in the topography image^60^. All images were nominally corrected for tilt and filtered using 3 × 3 Gaussian filter in the SPIP (Image Metrology A/S) image processing software.

### Transmission electron microscopy

Negatively stained preparations were made by adsorbing the control or protein-added MTs onto freshly glow-discharged carbon coated copper grids. The grids were washed and stained with 0.25% (w/v) uranyl acetate for 20 s, wicked off, and then washed with Millipore H_2_O. The samples were allowed to dry overnight before measurements. Images of negatively stained MTs were captured on a FEI Tecnai TF20 microscope operating at 200 kV with a nominal magnification of × 29,000 and × 50,000, respectively.

### Statistical analysis

Data are presented as mean± s.e.m. The normality of the data was assessed using the Shapiro-Wilk test. The normally distributed data were analysed with modified Student's (Welch) *t*-test at the 99% confidence level. All data analyses were performed using R software. The data were plotted using Origin 8, Minitab or GraphPad Prism 6 software. The figures were organized using Adobe Photoshop and Adobe Illustrator.

### Data availability statement

All relevant data are available from the authors.

## Additional information

**How to cite this article:** Thomas, G. E. *et al*. EB1 regulates attachment of Ska1 with microtubules by forming extended structures on the microtubule lattice. *Nat. Commun.* 7:11665 doi: 10.1038/ncomms11665 (2016).

## Supplementary Material

Supplementary Information Supplementary Figures 1-7, Supplementary Table 1, Supplementary Methods and Supplementary References

Supplementary Movie 1 Spatial positioning of EB1 and Ska1-GFP localization in Ska-GFP HeLa cell.

Supplementary Movie 2 Spatio-temporal visualization of the movements of the kinetochores attached with the kinetochore microtubules in a control Luc siRNA-treated Cos-7 cell.

Supplementary Movie 3 Spatio-temporal visualization of the movements of the kinetochores attached with the kinetochore microtubules in an EB1 siRNA-treated Cos-7 cell.

Supplementary Movie 4 Spatio-temporal visualization of the movements of the kinetochores attached with the kinetochore microtubules in a Ska1 siRNA-treated Cos-7 cell.

Supplementary Movie 5 Spatio-temporal visualization of the movements of the kinetochores attached with the kinetochore microtubules in an EB1 siRNA, Ska1 siRNA-treated Cos-7 cell.
