# Supplementary material for: EB1 regulates attachment of Ska1 with microtubules by forming extended structures on the microtubule lattice
Source: Nat Commun. 2016 May 26;7:11665. doi: 10.1038/ncomms11665 (PMC4894954; doi:10.1038/ncomms11665)
Supplement: Supplementary Information — Supplementary Figures 1-7, Supplementary Table 1, Supplementary Methods and Supplementary References [file ncomms11665-s1.pdf]

Supplementary Figure 1

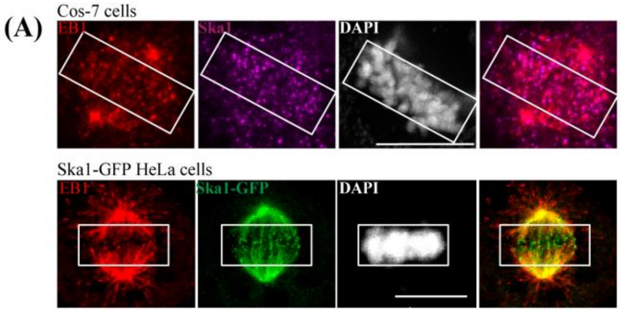

n=25	Correlation coefficient
EB1-Ska1	0.415 ± 0.013
EB1-Ska1 GFP	0.413 ± 0.018

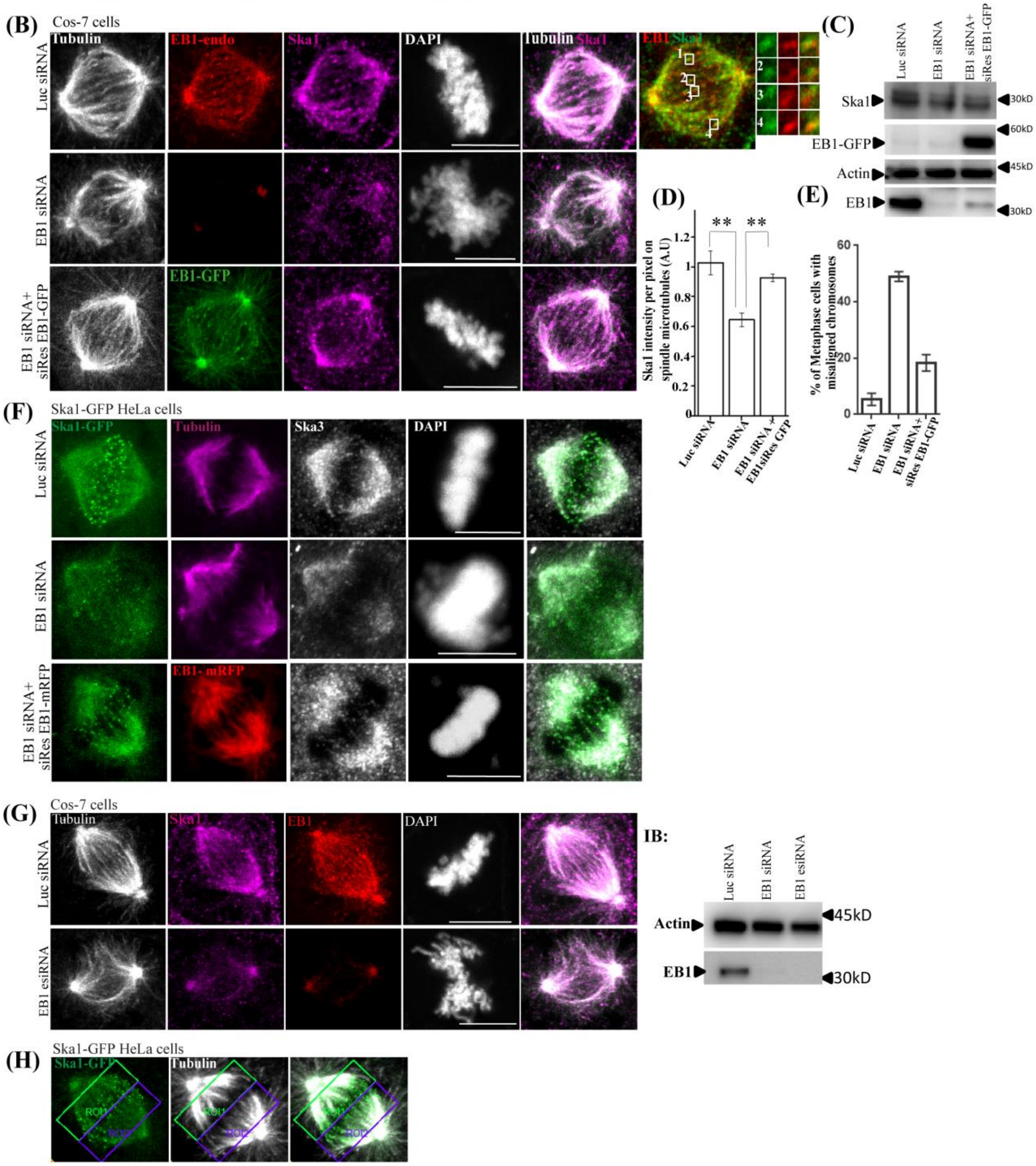

Supplementary Fig. 1 A) Representative confocal images of Cos-7 and Ska1-GFP HeLa cells showing the ROI selection for the colocalization analysis of EB1 and Ska1 on the microtubules on both sides of the metaphase plate. Scale bar =10 µm. Table shows the correlation coefficients of EB1 and Ska1 colocalization in Cos-7 and Ska1-GFP HeLa cells. B) Representative confocal images of mitotic Cos-7 cells transfected with control luciferase siRNA (48 hrs) or EB1 siRNA or EB1 siRNA + siRNA resistant EB1-GFP construct (siRes EB1-GFP). Microtubules (white), EB1 (red) and Ska1 (pink) were stained with antibodies. Chromosomes were stained with DAPI (shown in white). Scale bar =10 µm. Images in the extreme right side show enlarged view of localization of Ska1 (green) and EB1 (red) in control cells. In bottom panel, expression of exogenous siRes EB1-GFP is shown (green). C) Western blot of Cos-7 cell lysates showing endogenous EB1, Ska1 and siRes EB1-GFP at different conditions. Actin was probed as a control. D) Plot shows quantification of Ska1 intensity on spindle microtubules in Cos-7 cells at specified conditions, representation of regions selected for quantification is shown in Supplementary Fig. 1H, method of analysis is provided in 'Methods' (no of cells, n = 25), *** $=p<10^{-10}$, ** = $p< 10^{-5}$. Data are mean +/- s.e.m. E) Quantification of mitotic metaphase cells with misaligned chromosomes at specified conditions (no of cells counted, n = ~ 120). Data are mean +/- s.e.m. F) Confocal images showing Ska3 localization in Ska1-GFP HeLa cells transfected with luciferase siRNA, or EB1 siRNA or EB1 siRNA + siResEB1-mRFP, scale bar =10 µm. G) Confocal images of luciferase esiRNA- and EB1 esiRNA-treated Cos-7 cells stained for microtubules (white), EB1 (red) and Ska1 (pink), scale bar =10 µm. Western blot shows the levels of EB1 expression in control luciferase siRNA, EB1 siRNA and EB1 esiRNA-treated Cos -7 cells after 48 h of transfection. H) Representative image showing the ROI used for intensity analysis of Ska1-GFP on the microtubules on both sides of the metaphase plate in Ska1-GFP

HeLa cells. Similar ROIs were selected in Cos-7 cells under different treatment conditions as specified.

Supplementary Figure 2

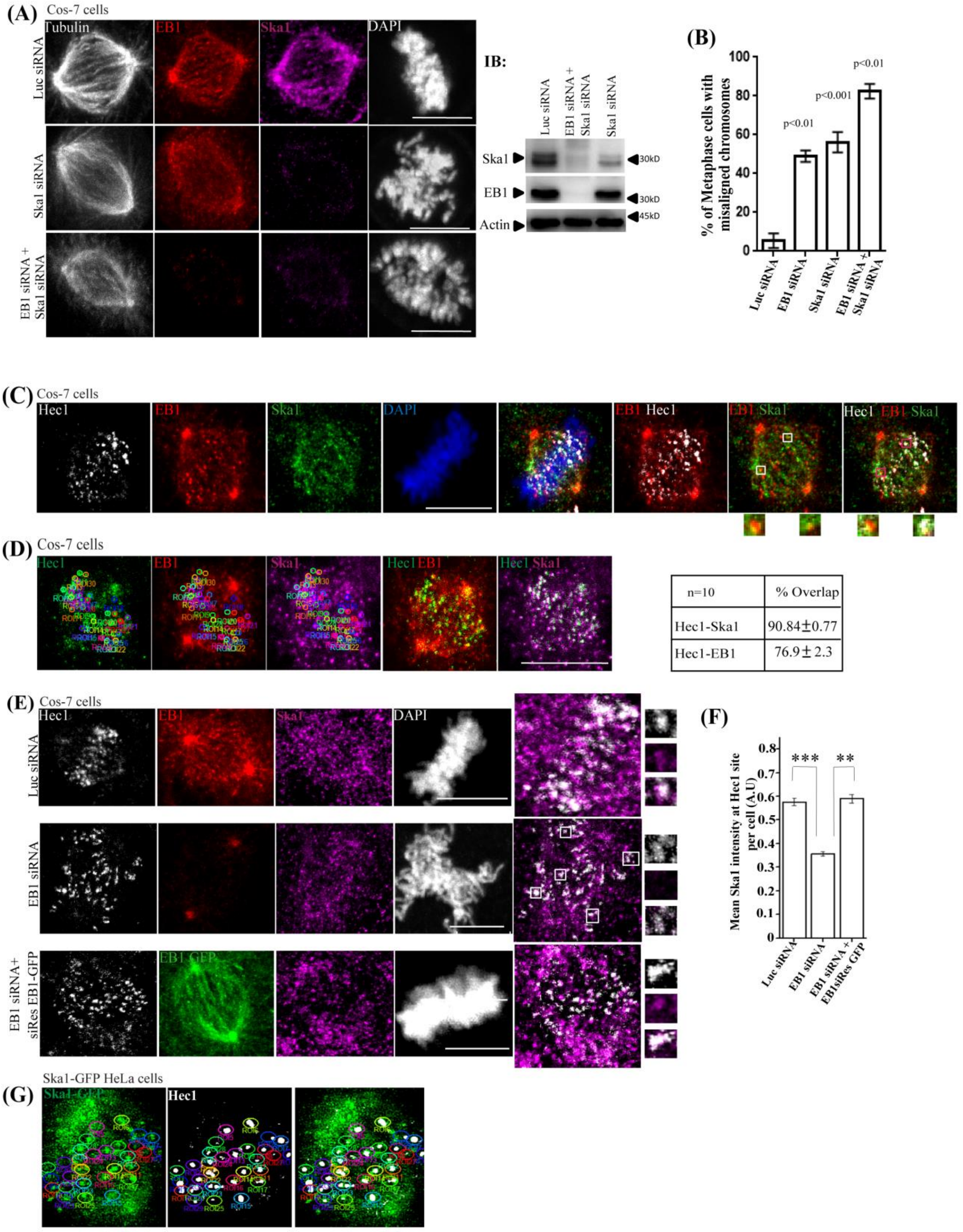

Supplementary Fig. 2 A) Confocal images of Ska1-depleted and Ska1, EB1 co-depleted Cos-7 cells stained for microtubules (white), EB1 (red) and Ska1 (pink), scale bar =10 µm. Western blot image shows levels of depletion of EB1 and Ska1 after 48 h of siRNA transfection. B) Plot shows the percentage of metaphase cells with misaligned chromosomes in control vs. other conditions as indicated (no. of cells = ~ 120). Data are mean +/- s.e.m. C) Representative confocal images of a metaphase Cos-7 cell showing the localization of Hec1 (white), EB1 (red) and Ska1 (green). Scale bar: 10µm. Enlarged views of Ska1, Hec1 and EB1 colocalization are shown in insets. Colocalization parameters are provided in Fig. S2D and the methods of analysis are provided in 'Methods'. D) Representative control Cos-7 cell images showing the regions of interest (ROI) selected for measurement of colocalization of Hec1, Ska1 and Hec1, EB1. The table shows the colocalization parameters of the proteins in Cos-7 and Ska1-GFP HeLa cells. E) Luciferase siRNA- or EB1-siRNA-transfected Cos-7 cells were immunostained for Ska1 (pink) and Hec1 (white). EB1 is shown in red. Bottom panel shows images of cells co-transfected with EB1-siRNA and siRes EB1-GFP (green). Scale bar =10 µm. For better view of Ska1 localization at Hec1 sites, enlarged images and boxes are shown on the right. F) Plot shows the quantification of mean Ska1 intensities at Hec1 sites at different treatment conditions as indicated, in Cos-7 cells. (n= ~ 25). ** $=p<10^{-5}$, data are mean +/- s.e.m. G) Representative image showing the ROI used for intensity analysis of Ska1-GFP at the individual Hec1 sites in the control and different treatment conditions in Ska1-GFP HeLa cells. Similar ROIs were selected in Cos-7 cells under different treatment conditions as specified.

Supplementary Figure 3

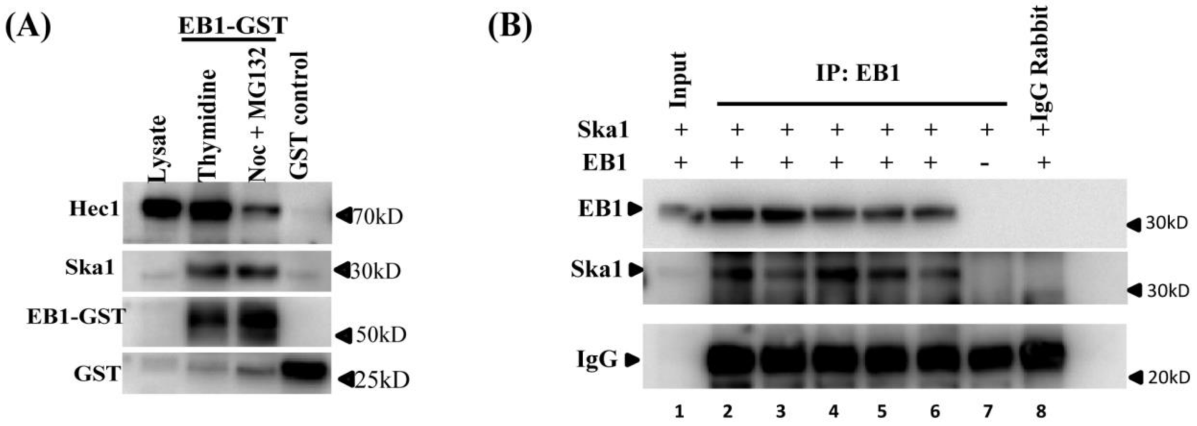

Supplementary Fig. 3. A) Lysate of thymidine-released synchronized mitotic Cos-7 cells with or without post-treatment of nocodazole (3.3 µM) and MG-132 (5 µM) was incubated with GST-tagged EB1 and subjected to GST-pull down using glutathione-Sepharose beads. Ska1 and Hec1 associated with EB1-GST were detected by Western blot. Control experiment was performed with the thymidine-released mitotic cell lysate by using purified GST (last lane). Amount of Ska1 and Hec1 pulled-down with EB1-GST were reduced by ~30 and 70%, respectively in the nocodazole- treated condition compared with the absence of nocodazole (two experiments). B) Mixtures of purified recombinant EB1 (5 µM) and Ska1 (10, 5, 2.5, 1 and 0.5 µM) were subjected to immunoprecipitation (IP) using EB1 antibody. EB1-IP in the reaction mixture containing only Ska1 (1 µM) (lane 7) is shown as a negative control.

Supplementary Figure 4

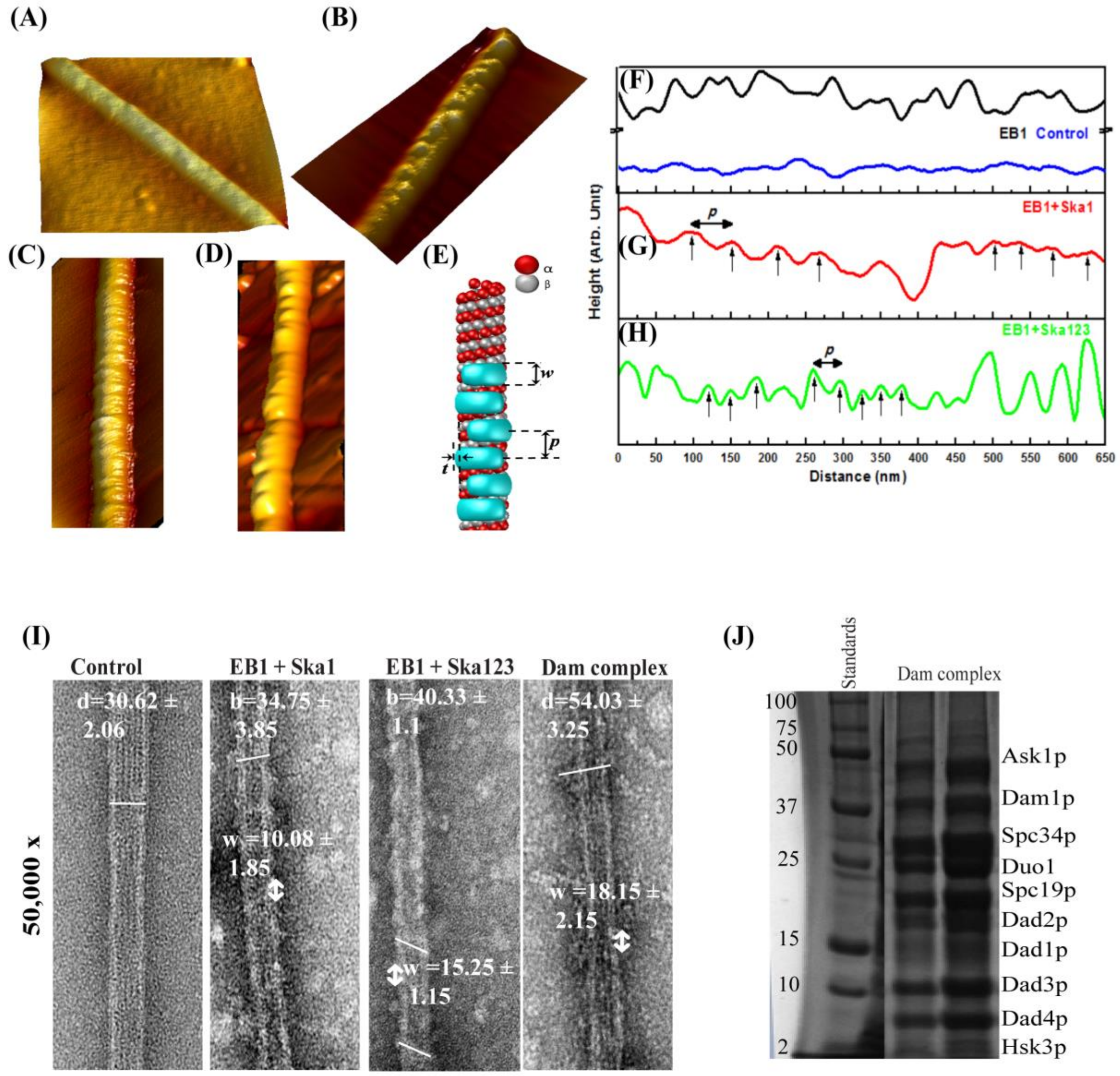

Supplementary Fig. 4 A) 3D AFM topography image of (A) control (bare) microtubule and microtubule treated with (B) EB1, showing globular structures on the microtubule lattice; (C) EB1-Ska1 and (D) EB1-Ska complex, both C and D showing binding structures extending along the girth of the microtubule lattice. (E) A cartoon depicting the formation of the extended structures on the microtubules seen in (C) and (D) and the parameters, thickness (t), width (w) and periodicity (p) used for quantifying the structures. (F-H) Line scans taken along the lengths

of microtubules shown in (A – D); (F) bare microtubule (blue line) and EB1-treated (black line), (G) EB1-Ska1 treated (red line) and (H) EB1-Ska treated (green line). I) Images of TEM showing the diameter (d) and width (w) of bare microtubule or the Dam1/DASH rings on the microtubule; or the breadth (b) and the width (w) of the microtubule binding structures formed by either EB1-Ska1 or EB1-Ska. J) Gel image showing purified recombinant Dam1/DASH complex proteins run on SDS-PAGE followed by Coomassie staining.

Supplementary Figure 5

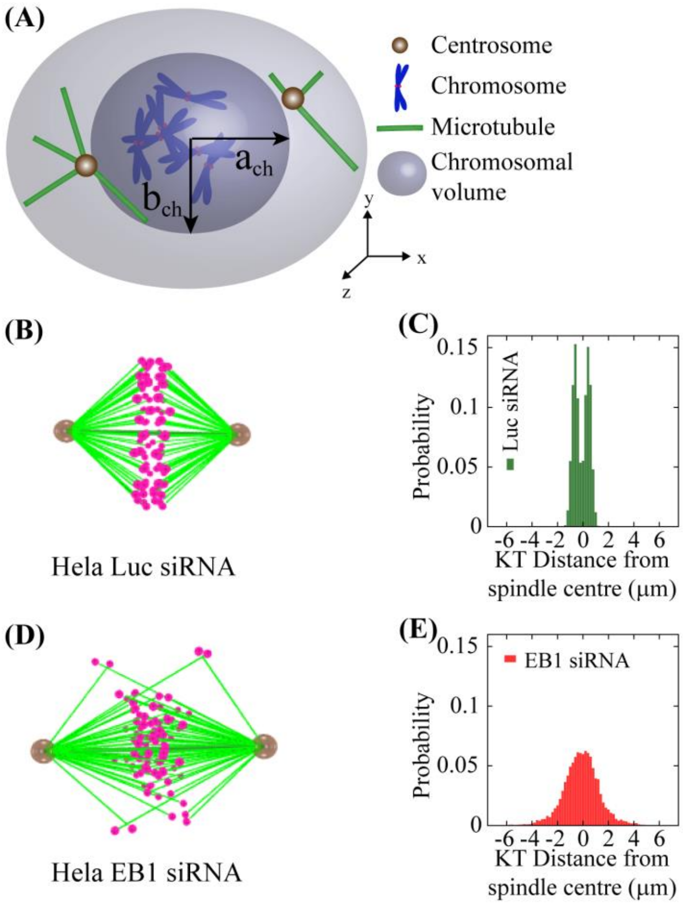

Supplementary Fig. 5 A) Cartoon of the simulation of the simulation system showing the chromosomal territory and the position of centrosomes in the beginning of simulation. a_{ch} and b_{ch} are the two axes of the cross-section of the chromosomal volume. B and C represent the simulation snapshot of chromosomal configuration and distribution, respectively around the metaphase plate in Luc siRNA-treated control Ska1-GFP HeLa cells. Similar to Cos-7 cells, this snapshot of *in silico* phenotypes show perfectly aligned chromosomes around the metaphase plate. D and E correspond to the snapshot of chromosomal configuration and distribution,

respectively in EB1-depleted Ska1-GFP HeLa cells. The distribution of chromosomes show dispersed chromosomes around the metaphase plate.

Supplementary Figure 6

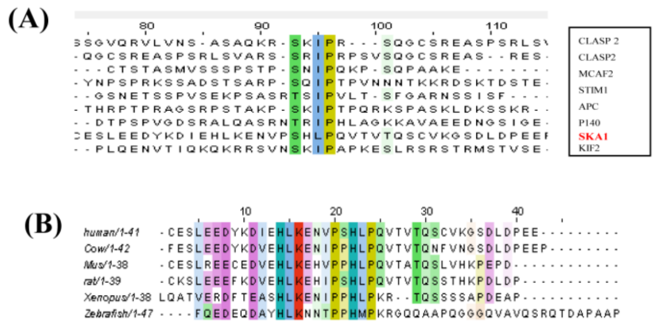

Supplementary Fig. 6 A) Sequence alignment of EB1-binding human +TIP proteins with Ska1 showing SHLP motif similar to SxIP motif present in +TIPs (B) Conservation of Ska1 SHLP motif across various species is shown. Alignments were performed by CLUSTAL W.

Supplementary Figure 7

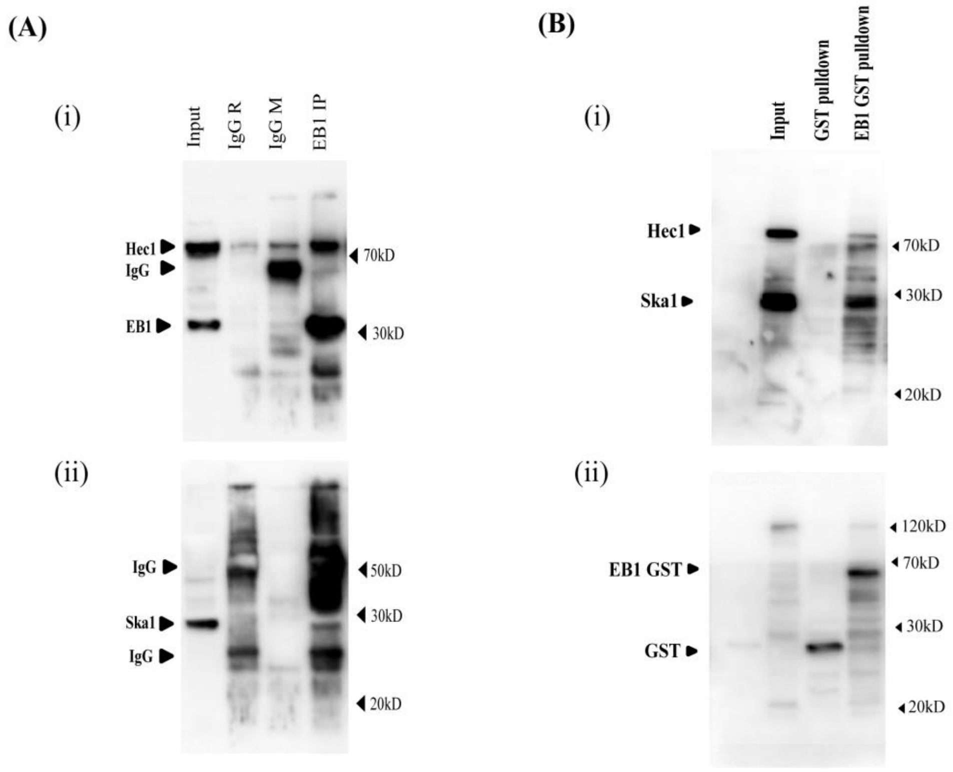

Supplementary Fig. 7. Un-cropped scans of the Western blots corresponding to the data shown in (A) Fig. 2D and (B) Fig. 2F. In i) of A: Co-IP of EB1, the blot probed for Hec1 and EB1 with respective mouse monoclonal antibodies of Hec1 and EB1 and in ii) the same probed for Ska1 with respective rabbit polyclonal antibody of Ska1. In i) of B, GST pull down samples were run and the blot was probed for Ska1 and Hec1 with the respective rabbit polyclonal and mouse monoclonal antibody, and in ii) the blot was probed for GST and GST-EB1 by GST-specific antibody.

Supplementary Table 1. Various parameters used to develop the model

Abbreviations	Meaning	Values for used in the model	Reference
N_{KT}	Number of chromosomes	~46 (HeLa)<br>~60 (Cos-7)	Our experiment
a_{cell} ,b_{cell},c_{cell}	Dimension of the Cell	10, 10, 10 μm (HeLa)<br>12, 10, 10 μm (Cos-7)	Our experiment
A	Strength of the polar ejection close to the pole	25	[22]
L	Average length of the microtubule	3 (Luc siRNA)<br>2 (EB1 siRNA)	[22]
K_{cor}	Spring constant of the cortex region	5.0 pN/μm	[43]
r_{KT}	Radius of single KT	0.5 μm	[44]
v_g, v_s	growth, shrink velocity of MT	0.25 μm sec^{-1}, 0.4 μm sec^{-1}	[45-47]
f_c,f_r	catastrophe, rescue frequency of MT	0.34 min^{-1}, 0.02 min^{-1} (Luc siRNA)<br>0.15 min^{-1}, 0.02 min^{-1} (EB1 siRNA)	[48]
f_s	Stall force of MT	1.7 pN	[38]
f_{dyn}^{s}	Force produced by single dynein	1.0 pN	[49, 50]
λ_{dyn}	Density of dynein per unit length per MT	6.0 μm	[45]
λ_{ipMT}	Density of ipMT motors per unit length	5.0 μm	[45]
α	Force produced by KT motors per unit length	1.0 pN	This study
ξ	coefficient of viscosity of cytoplasm	5.0 pN s/μm^2	[45]
$K_{cohesion}$	Spring constant of the cohesion springs	10pN/μm	[47]
K_C	Spring constant of the Ndc80 springs	10.0 pN/μm(Luc siRNA)<br>5.0 pN/μm (EB1siRNA)	[45, 51]
$K_{fibrils}$	Spring constant of the KT fibrils	2.50 pN/μm	[45, 51]

Supplementary Methods

Here we describe the model variables and governing equations (Fig. 6 A) to understand the mechanistic roles of EB1-Ska1/Ska complex on the stability of microtubule-kinetochore attachment. We construct a simple three dimensional mechanistic model to observe the effect on the spindle formation and chromosome configuration. Our model contains two centrosomes and N number of chromosomes. In the beginning of the simulation, chromosomes are distributed randomly within a spheroidal volume centered at (0, 0, 0) with semi-major axes $a_{ch,}$ b_{ch} and c_{ch} along the x, y and z-axes, respectively, as shown in Fig. S5 A. This ensures the chromosomal localization within a spheroidal nuclear volume prior to the nuclear envelope breakdown (NEB). We further assume that, initially the centrosomes are localized on the surface of the nuclear envelope in a diametrically opposite position. Upon NEB, centrosomal MTs enter the nuclear sphere and form the spindle. A section of centrosomal microtubules interact with the chromosome arms and generate polar ejection force away from the poles[1-5], while another section 'search and capture' the kinetochores (KT) [6-9] attaching the chromosomes with the spindle. Kinetochore-microtubules (kMTs) generate pole-ward tension on the KT[10-14], whereas astral MTs pull the centrosome toward the cell periphery[15-17]. Inter-polar MTs (ipMT) form anti-parallel overlap at the equatorial region of the spindle and slide against each other[18-20]. Majority of these interactions generate forces that are exerted by molecular motors. The formation of a stable bipolar spindle is a concerted effort of all the interactions outlined above. In our *in silico* framework, these forces have been incorporated in the following manner:

Polar ejection force: Polar ejection force is a function of the distance between the pole and KT. We assume that MTs nucleated at the poles exhibit an exponentially decaying length distribution. Using this exponential form, one can write the number of MTs at a distance x from the pole as

$N(x) \propto \exp(-x/L)$ [21, 22] and calculate the polar ejection force experienced by a single chromosome as:

$$F^{i}_{ejection} = A\exp(-(x/L) \quad (1)$$

where, A is the maximal polar ejection force and L is the average MT length. Values of A and L were chosen from experimental data (See Table 1).

Pole-KT attraction: The dynamics of the kMT plus end plays a crucial role in positioning the KT[23-27]. kMTs interact with the inner KT through spring-like KT fibrils [28]. As the polymerizing kMT tip penetrates the KT, it applies a pushing force on the KT following $F_{\text{poly}} = l_{\text{pen}}K_{\text{fibrils}}$, where l_{pen} is the length of penetration of the kMT tip within the KT and K_{fibrils} is the effective spring constant of the KT fibrils. A depolymerizing kMT pulls the KT with a force $F_{\text{depoly}}=K_{\text{c}}l_{\text{out}}$ upon exiting the KT. Here, l_{out} is the separation between the kMT tip and the KT and K_{c} is the stiffness constant of the kMT-KT connecting springs mimicking the interaction between Ndc80 and EB1-Ska1 complex [29, 30]. To maintain a stable attachment between KT and kMT, the depolymerizing kMT is rescued with the frequency: f_{r}=1-exp ($-F_{\text{depoly}}/f_{\text{s}}$), where, f_{s} is the stall force of MT. We hypothesized that, upon Ska1 or EB1 depletion, the interaction between the kMT and Ndc80 becomes weaker, which eventually leads to smaller rescue of kMT. Several molecular motors like CENPE, dynein have been known to be localized at the KT generating poleward force[12, 13, 31-33]. We model the molecular motor-driven forces at the KT as $F_{\text{motor}}=\alpha l_{\text{in}}$, where α represents the force generated by the molecular motors per unit length and l_{in} is the length of the kMT within the KT. It is widely believed that each KT interacts with a large number of kMTs (~10-30) in mammalian cells. Therefore, we calculate the total force acting between the centrosome and a single KT as:

$$F_{\mathrm{kMT}} = \sum(F_{\mathrm{poly}} + F_{\mathrm{depoly}} + F_{\mathrm{motor}}) \quad (2)$$

where, the sum is over the number of kMTs interacting with a single KT. The kMTs are modeled as straight filaments and dynamically evolving with growth and shrinkage rates v_g and v_s, respectively. Stochastic turnover and recovery of MTs are achieved by regulating catastrophe and rescue frequencies, f_c and f_r, respectively[34] .

Cohesion between sister-chromatids: The sister KTs remain attached to each other by cohesion springs. These cohesion springs, when stretched, generate tension between the sister-KTs:

$$F_{\mathrm{cohesion}} = K_{\mathrm{cohesion}} x_{\mathrm{KT}} \quad (3)$$

where, K_{cohesion} is the spring constant of the cohesion springs and x_{KT} is the separation between the sister-KTs.

Pulling from cortex by astral microtubules: Astral microtubules (cMTs) interact with the cell cortex primarily via dynein motors [35-37]. We assume that the cortex region acts as a static wall that resists the growth of a cMT. The growth velocity of a cMT within the cortical region decreases as $v_g = v_g^0 \exp(-K_{\mathrm{cor}} l_{\mathrm{dyn}}/f_s)$ [38, 39], where v_g^0 is the growth velocity of a free MT, K_{cor} is the stiffness of the cortex, l_{dyn} is the length of penetration of the cMT tip within the cortex region and f_s is the stall force per MT. As the tip of a cMT grows within the cortical region, dynein binds to it and pulls the centrosome toward the cortex[35-37, 40]. The pulling force exerted by dynein is calculated using the following expression:

$$f_{\mathrm{dyn}}^{\mathrm{s}} = l_{\mathrm{dyn}} \lambda_{\mathrm{dyn}} f_{\mathrm{dyn}} \quad (4)$$

Here, λ_{dyn} is the dynein density per unit length and f_{dyn}^{s} is the magnitude of the force exerted by a single dynein motor. Summing f_{dyn} over all the cMTs, we get the resultant pulling force F_{dyn} on

the centrosome. The cMTs also exert a net pushing force F_{push}, when their tips hit the cell periphery. The pushing force is primarily due to the polymerization of the cMT tip in contact with the cell cortex. The total force due the cortex- cMT interaction is given by

$F_{\text{cortex}} = F_{\text{dyn}} + F_{\text{push}}$.

Sliding force between inter-polar MTs: Due to the actions of kinesin 5 motors along the ipMT overlap [41, 42], sliding MTs push the centrosomes away from each other. The pushing force F_{ipMT} reads as:

$$F_{\text{ipMT}} = l_{\text{overlap}} \lambda_{\text{ipMT}} f_{\text{ipMT}} \qquad (5)$$

where, l_{overlap} is the total overlap length among all the ipMTs nucleated from the opposite centrosomes, λ_{ipMT} is the linear density of kinesin motors along the ipMTs and f_{ipMT} is the force produced by a single kinesin motor.

Collecting all these forces, mentioned above (Equations 1- 5), the equation of motion of the ith KT/chromosome and the centrosome can now be written as:

$$\frac{dx_{KT}^{i}}{dt} = \frac{F_{kMT}^{i} + F_{cohesion}^{i} + F_{ejection}^{i}}{\xi_{KT}^{i}} \qquad (6)$$

$$\frac{dx_{cen}}{dt} = \frac{\sum_{i=1}^{N_{KT}} (F_{kMT}^{i} + F_{ejection}^{i}) + F_{ipMT} + F_{cortex}}{\xi_{cen}} \qquad (7)$$

Here, x's represent the instantaneous coordinates of KT and centrosome. The system of equations (Eq. 7 and 8) are derived in accordance with well-known Stokes law $v = F/\xi$; v, F and ξ being the velocity, force and viscous drag of a moving object. The viscous drag obeys the formula $\xi = 6\pi\eta r$, where η is the coefficient of viscosity of the medium and r is the effective

radius of the object. Here ξ_{KT} and ξ_{cen} correspond to the effective drag on a KT and on a centrosome, respectively. At each time step, all the forces are calculated using Eq. 1 – 5 and then Eqs. 6 and 7 are solved numerically to update the positions of KTs and centrosomes. We explore a range of values for the model parameters (**Table 1**) to evaluate the quality and predictability of the model.

Supplementary References

1. Antonio, C. *et al.* Xkid, a chromokinesin required for chromosome alignment on the metaphase plate. *Cell* **102**, 425-435 (2000).
2. Brouhard, G.J. & Hunt, A.J. Microtubule movements on the arms of mitotic chromosomes: polar ejection forces quantified in vitro. *Proc. Natl. Acad. Sci., USA* **102**, 13903-13908 (2005).
3. Ke, K., Cheng, J. & Hunt, A.J. The distribution of polar ejection forces determines the amplitude of chromosome directional instability. *Curr. Biol .* **19**, 807-815 (2009).
4. Levesque, A.A. & Compton, D.A. The chromokinesin Kid is necessary for chromosome arm orientation and oscillation, but not congression, on mitotic spindles. *J. Cell. Biol.* **154**, 1135-1146 (2001).
5. Rieder, C.L. & Salmon, E.D. Motile Kinetochores and Polar Ejection Forces Dictate Chromosome Position on the Vertebrate Mitotic Spindle. *J. Cell Biol.* **124**, 223-233 (1994).
6. Hayden, J.H., Bowser, S.S. & Rieder, C.L. Kinetochores Capture Astral Microtubules during Chromosome Attachment to the Mitotic Spindle: Direct Visualization in Live Newt Lung Cells. *J. Cell Biol.* **111**, 1039-1045 (1990).
7. Kalinina, I. *et al.* Pivoting of microtubules around the spindle pole accelerates kinetochore capture. *Nat. Cell Biol.* **15**, 82-87 (2013).
8. Paul, R. *et al.* Computer simulations predict that chromosome movements and rotations accelerate mitotic spindle assembly without compromising accuracy. *Proc. Natl. Acad. Sci. USA* **106**, 15708-15713 (2009).
9. Wollman, R. *et al.* Efficient chromosome capture requires a bias in the 'search-and-capture' process during mitotic-spindle assembly. *Curr. Biol .* **15**, 828-832 (2005).
10. Cheeseman, I.M., Chappie, J.S., Wilson-Kubalek, E.M. & Desai, A. The conserved KMN network constitutes the core microtubule-binding site of the kinetochore. *Cell* **127**, 983-997 (2006).
11. Cheeseman, I.M. & Desai, A. Molecular architecture of the kinetochore-microtubule interface. *Nat. Rev Mol. Cell Biol.* **9**, 33-46 (2008).
12. Li, Y., Yu, W., Liang, Y. & Zhu, X. Kinetochore dynein generates a poleward pulling force to facilitate congression and full chromosome alignment. *Cell Res.* **17**, 701-712 (2007).
13. Pfarr, C.M. *et al.* Cytoplasmic dynein is localized to kinetochores during mitosis. *Nature* **345**, 263-265 (1990).
14. Rieder, C.L. & Salmon, E.D. The vertebrate cell kinetochore and its roles during mitosis. *Trends Cell Biol.* **8**, 310-318 (1998).
15. Adames, N.R. & Cooper, J.a. Microtubule interactions with the cell cortex causing nuclear movements in Saccharomyces cerevisiae. *J. Cell. Biol.* **149**, 863-874 (2000).

16. Carminati, J.L. & Stearns, T. Microtubules orient the mitotic spindle in yeast through dynein-dependent interactions with the cell cortex. *J. Cell Biol.* **138**, 629-641 (1997).
17. Fink, G., Schuchardt, I., Colombelli, J., Stelzer, E. & Steinberg, G. Dynein-mediated pulling forces drive rapid mitotic spindle elongation in Ustilago maydis. *EMBO J.* **25**, 4897-4908 (2006).
18. Brust-Mascher, I., Civelekoglu-Scholey, G., Kwon, M., Mogilner, A. & Scholey, J.M. Model for anaphase B: role of three mitotic motors in a switch from poleward flux to spindle elongation. *Proc. Natl. Acad. Sci., USA* **101**, 15938-15943 (2004).
19. Mastronarde, D.N., McDonald, K.L., Ding, R. & McIntosh, J.R. Interpolar spindle microtubules in PTK cells. *J. Cell Biol.* **123**, 1475-1489 (1993).
20. Sharp, D.J. *et al.* The Bipolar Kinesin, KLP61F, Cross-links Microtubules within Interpolar Microtubule Bundles of. *J. Cell Biol.* **144**, 125-138 (1999).
21. Ferenz, N.P., Paul, R., Fagerstrom, C., Mogilner, A. & Wadsworth, P. Dynein antagonizes eg5 by crosslinking and sliding antiparallel microtubules. *Curr. Biol .* **19**, 1833-1838 (2009).
22. Sutradhar, S., Basu, S. & Paul, R. Intercentrosomal angular separation during mitosis plays a crucial role for maintaining spindle stability. *Phys Rev E Stat Nonlin Soft Matter Phys* **92**, 28 (2015).
23. Fernandez, N., Chang, Q., Buster, D.W., Sharp, D.J. & Ma, A. A model for the regulatory network controlling the dynamics of kinetochore microtubule plus-ends and poleward flux in metaphase. *Proc. Natl. Acad. Sci. USA* **106**, 7846-7851 (2009).
24. Gardner, M.K. *et al.* Tension-dependent Regulation of Microtubule Dynamics at Kinetochores Can Explain Metaphase Congression in Yeast â–¡. *Mol. Biol. Cell* **16**, 3764-3775 (2005).
25. Gorbsky, G.J., Sammak, P.J. & Borisy, G.G. Chromosomes move poleward in anaphase along stationary microtubules that coordinately disassemble from their kinetochore ends. *J. Cell Biol.* **104**, 9-18 (1987).
26. Hyman, A.A. & Mitchison, T.J. Modulation of microtubule stability by kinetochores in vitro. *J. Cell Biol.* **110**, 1607-1616 (1990).
27. Waters, J.C., Mitchison, T.J., Rieder, C.L. & Salmon, E.D. The kinetochore microtubule minus-end disassembly associated with poleward flux produces a force that can do work. *Mol. Biol. Cell* **7**, 1547-1558 (1996).
28. McIntosh, J.R. *et al.* Fibrils connect microtubule tips with kinetochores: a mechanism to couple tubulin dynamics to chromosome motion. *Cell* **135**, 322-333 (2008).
29. Powers, A.F. *et al.* The Ndc80 kinetochore complex forms load-bearing attachments to dynamic microtubule tips via biased diffusion. *Cell* **136**, 865-875 (2009).
30. Wei, R.R., Al-Bassam, J. & Harrison, S.C. The Ndc80/HEC1 complex is a contact point for kinetochore-microtubule attachment. *Nat Struct Mol Biol* **14**, 54-59 (2007).
31. Schaar, B.T., Chan, G.K., Maddox, P., Salmon, E.D. & Yen, T.J. CENP-E function at kinetochores is essential for chromosome alignment. *J. Cell Biol.* **139**, 1373-1382 (1997).
32. Wood, K.W., Sakowicz, R., Goldstein, L.S. & Cleveland, D.W. CENP-E is a plus end-directed kinetochore motor required for metaphase chromosome alignment. *Cell* **91**, 357-366 (1997).
33. Cytrynbaum, E.N., Scholey, J.M. & Mogilner, A. A force balance model of early spindle pole separation in Drosophila embryos. *Biophys. J.* **84**, 757-769 (2003).
34. Mitchison, T. & Kirschner, M. Dynamic instability of microtubule growth. *Nature* **312**, 237-242 (1984).
35. Adames, N.R. & Cooper, J.A. Microtubule interactions with the cell cortex causing nuclear movements in *Saccharomyces cerevisiae*. *J Cell Biol* **149**, 863-874 (2000).
36. Carminati, J.L. & Stearns, T. Microtubules orient the mitotic spindle in yeast through dynein-dependent interactions with the cell cortex. *J Cell Biol* **138**, 629-641 (1997).

37. Ten Hoopen, R. *et al.* Mechanism for astral microtubule capture by cortical Bud6p priming spindle polarity in *S. cerevisiae*. *Curr Biol* **22**, 1075-1083 (2012).
38. Dogterom, M. & Yurke, B. Measurement of the force-velocity relation for growing microtubules. *Science* **278**, 856-860 (1997).
39. Janson, M.E., de Dood, M.E. & Dogterom, M. Dynamic instability of microtubules is regulated by force. *J Cell Biol* **161**, 1029-1034 (2003).
40. Lee, L. *et al.* Positioning of the mitotic spindle by a cortical-microtubule capture mechanism. *Science* **287**, 2260-2262 (2000).
41. Kapoor, T.M. & Mitchison, T.J. Eg5 is static in bipolar spindles relative to tubulin: evidence for a static spindle matrix. *J Cell Biol* **154**, 1125-1133 (2001).
42. Marco, E. *et al. S. cerevisiae* chromosomes biorient via gradual resolution of syntely between S phase and anaphase. *Cell* **154**, 1127-1139 (2013).
43. Sutradhar, S. *et al.* A comprehensive model to predict mitotic division in budding yeasts. *Mol Biol Cell* **26**, 3954-3965 (2015).
44. Haase, J. *et al.* A 3D map of the yeast kinetochore reveals the presence of core and accessory centromere-specific histone. *Current biology : CB* **23**, 1939-1944 (2013).
45. Civelekoglu-Scholey, G., Sharp, D.J., Mogilner, A. & Scholey, J.M. Model of chromosome motility in Drosophila embryos: adaptation of a general mechanism for rapid mitosis. *Biophysical journal* **90**, 3966-3982 (2006).
46. Foethke, D., Makushok, T., Brunner, D. & Nedelec, F. Force- and length-dependent catastrophe activities explain interphase microtubule organization in fission yeast. *Molecular systems biology* **5**, 241 (2009).
47. Joglekar, A.P. & Hunt, A.J. A simple, mechanistic model for directional instability during mitotic chromosome movements. *Biophysical journal* **83**, 42-58 (2002).
48. Gregoretti, I.V., Margolin, G., Alber, M.S. & Goodson, H.V. Insights into cytoskeletal behavior from computational modeling of dynamic microtubules in a cell-like environment. *J. Cell Sci.* **119**, 4781-4788 (2006).
49. Soppina, V., Rai, A.K., Ramaiya, A.J., Barak, P. & Mallik, R. Tug-of-war between dissimilar teams of microtubule motors regulates transport and fission of endosomes. *Proceedings of the National Academy of Sciences of the United States of America* **106**, 19381-19386 (2009).
50. Muller, M.J., Klumpp, S. & Lipowsky, R. Tug-of-war as a cooperative mechanism for bidirectional cargo transport by molecular motors. *Proceedings of the National Academy of Sciences of the United States of America* **105**, 4609-4614 (2008).
51. Sau, S., Sutradhar, S., Paul, R. & Sinha, P. Budding yeast kinetochore proteins, Chl4 and Ctf19, are required to maintain SPB-centromere proximity during G1 and late anaphase. *PloS one* **9**, e101294 (2014).
